# Multi-dimensional data-driven computational drug repurposing strategy for screening novel neuroprotective agents in ischemic stroke

**DOI:** 10.7150/thno.112608

**Published:** 2025-06-23

**Authors:** Qingqi Meng, Qing Liu, Yan Mi, Libin Xu, Feng Wang, Danyang Mu, Yueyang Liu, Yuxin Yang, Yongye Huang, Dakuo He, Yue Hou

**Affiliations:** 1 Key Laboratory of Bioresource Research and Development of Liaoning Province, College of Life and Health Sciences, National Frontiers Science Center for Industrial Intelligence and Systems Optimization, Key Laboratory of Data Analytics and Optimization for Smart Industry, Ministry of Education, Northeastern University, Shenyang, China.; 2 College of Information Science and Engineering, State Key Laboratory of Synthetical Automation for Process Industries, Northeastern University, Shenyang, China.; 3 Shenyang Key Laboratory of Vascular Biology, Science and Research Center, Department of Pharmacology, Shenyang Medical College, Shenyang, China.

**Keywords:** ischemic stroke, novel neuroprotective agents, computational drug repositioning, sulbutiamine, PDK2

## Abstract

**Background:** The complexity of biological systems and misconceptions about neuroprotection have hindered the development of neuroprotective drugs for ischemic stroke. This study aims to identify new neuroprotective agents by integrating ischemic stroke transcriptomics with neuronal protection data using a Multidimensional Data-Driven Computational Drug Repositioning strategy (MDCDR).

**Methods**: Three microarray datasets related to ischemic stroke (GSE16561, GSE58294, and GSE22255) were obtained from the GEO dataset and pre - processed to analyze differentially expressed genes (DEGs). The Connectivity Map (CMap) database was used to predict potential drugs. A neuroprotection activity prediction model was constructed by combining six molecular fingerprints with three machine learning algorithms (Random Forest RF, Support Vector Machine SVM, Gradient Boosting Decision Tree GBDT) to screen for potential neuroprotective agents. The efficacy of the screened compounds was evaluated through* in vitro* experiments on SH-SY5Y cells treated with oxygen-glucose deprivation/reperfusion (OGD/R) and *in vivo* experiments on middle cerebral artery occlusion/reperfusion (MCAO/R) rat models. Multiple experimental techniques (such as RNA sequencing, DARTS, CETSA, etc.) were used to explore their potential mechanisms of action.

**Results**: The MDCDR strategy screened out 19 potential neuroprotective agents, among which sulbutiamine (SUL) stood out. SUL significantly increased the survival rate, reduced neurological deficit scores, and decreased neuronal loss in MCAO/R rat models, and inhibited cell death in OGD/R - induced cell models. Mechanistic studies revealed that SUL inhibited pyruvate dehydrogenase kinase 2 (PDK2), enhanced mitochondrial function, reduced reactive oxygen species (ROS) levels, thereby suppressing the MAPK signaling pathway and reducing neuronal apoptosis. Silencing PDK2 abolished the protective effect of SUL on OGD/R - treated SH - SY5Y cells.

**Conclusion**: This study successfully developed the MDCDR strategy for screening neuroprotective agents for ischemic stroke. SUL was identified as a promising neuroprotective agent, and PDK2 was a crucial target. This research provides new directions and a theoretical basis for the development of neuroprotective agents against ischemic stroke.

## Introduction

Ischemic stroke (IS), characterized by disrupted cerebral blood flow resulting from thrombosis or embolism, is a leading cause of mortality and disability worldwide 1-3. Thrombolytic therapy is currently necessary for the treatment of IS with its narrow therapeutic window and hemorrhagic risk 4,5. Neuroprotection is effective in ameliorating ischemia-reperfusion injury 6-8. Despite the evaluation of hundreds of potential neuroprotective agents in preclinical stroke studies over the past 25 years, only a handful have successfully transitioned to clinical use 6-8. This limited success underscores the challenges in translating promising findings into effective treatments. However, introducing the neurovascular unit concept and discovering new compounds with diverse mechanisms of action provide renewed optimism for advancing neuroprotection 6-8. Consequently, pursuing effective neuroprotective agents remains a critical priority in stroke research and therapeutic development.

Drug repurposing offers a cost-effective and time-efficient solution for drug development, especially with the emergence of machine learning technologies, which have become powerful tools for drug repositioning 9-15. For instance, Zhu et al. proposed an efficacy prediction system aimed at facilitating drug repurposing and discovery 16; Xie et al. utilized machine learning techniques to identify candidate drugs for Alzheimer's disease 17; and Stokes et al. trained deep neural networks to identify antibiotic drugs 18. However, these strategies often rely on single sources of information and fail to fully account for the complexity of biological systems. Therefore, in the development of strategies for novel neuroprotective drugs, leveraging machine learning methods to integrate diverse information holds promise as a new approach for identifying neuroprotective agents.

In this study, we amalgamated diverse aspects of disease information to develop the Multidimensional Data-driven Computational Drug Repositioning strategy (MDCDR). This strategy enables the screening of potential anti-IS neuroprotective agents from US Food and Drug Administration (FDA) approved drugs. Meanwhile, we used the SHapley Additive exPlanations (SHAP) interpreter to identify the key features that exert neuroprotective effects. Through a series of screenings, we identified new neuroprotective agents and established models of middle cerebral artery occlusion/reperfusion (MCAO/R) and oxygen-glucose deprivation/reperfusion (OGD/R) to evaluate the efficacy and mechanisms of the identified compounds. Additionally, we investigated the potential targets of these compounds through experimental methods and validated the potential application of these targets in IS.

## Materials and Methods

### Study design

**Figure 1:**
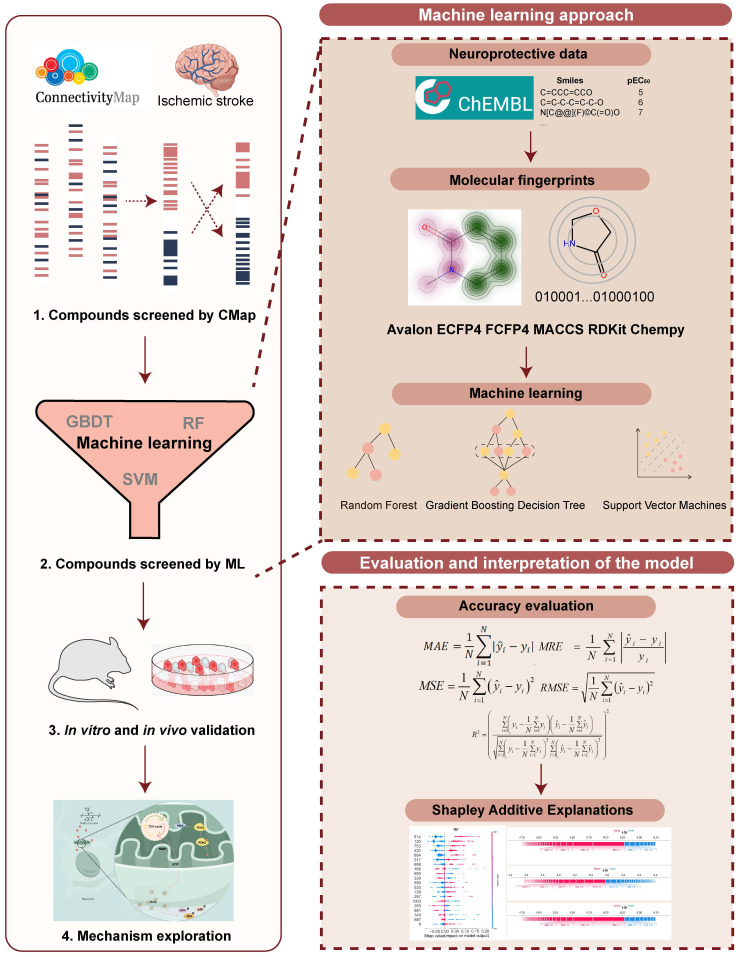
** The workflow of MDCDR.** The gene expression profiles of ischemic stroke were organized and the expression profiles were entered into the CMap database to screen drugs with anti-ischemic stroke potential. Marketed drugs with neuroprotective effects were screened using machine learning tools. The model is trained on 172 compounds with neuroprotective effects, utilizing 5 types of molecular fingerprints and 3 machine learning algorithms. Through evaluation methods such as MRE, the best-performing molecular fingerprints were selected to construct a consistent model. Finally, the SHAP method was employed to identify key features relevant to neuroprotective effects. The neuroprotective mechanisms of the compound were elucidated through in vitro and in vivo validation, transcriptome analysis, and mechanistic exploration.

A machine learning-based workflow for MDCDR was applied to screen neuroprotectants among FDA approved drugs (Figure 1). All abbreviations mentioned in the text are detailed in Supplementary Table S1. MDCDR consists of two modules. Firstly, we perform a genome-based analysis of the IS-associated compounds module. This involves analyzing the gene transcriptional profiles of IS using the Connectivity Map (CMap) platform, to identify compounds that potentially exhibit anti-IS properties. Next, we propose a neuroprotection activity prediction model (NPAPM) that integrates three powerful machine learning algorithms: Random Forest (RF), Support Vector Machine (SVM), and Gradient Boosting Decision Tree (GBDT). This part inputs the compounds screened in CMap into the integrated neuroprotection prediction model for screening compounds with neuroprotective effects. In addition, we evaluated the accuracy of the model using metrics such as mean relative error (MRE) and conducted an in-depth analysis of the model using the SHAP interpreter. Finally, the compounds with better-predicted results were selected for further pharmacodynamic and mechanistic studies in OGD/R exposed SH-SY5Y cells and MCAO/R rats, respectively.

### Datasets and pre-processing

This study obtained three microarray datasets related to IS from the GEO database (https://www.ncbi.nlm.nih.gov/geo/), and loaded these datasets using the GEOquery package in R (version 4.2.0). Specifically, the GSE16561 dataset contains 39 IS samples and 24 normal samples; the GSE58294 dataset contains 69 IS samples and 23 normal samples; the GSE22255 dataset contains 20 IS samples and 20 normal samples 19-21. To facilitate subsequent analysis, the gene expression levels were normalized by converting the signal intensities to Log2-transformed quantile-normalized values. Subsequently, probes were mapped to their corresponding genes, and uninformative probes were removed.

To identify differentially expressed genes (DEGs), the LIMMA (https://bioconductor.org/packages/release/bioc/html/limma.html) package was employed in this study. Specifically, we analyzed the normalized expression data using the linear model fitting function from the `limma` package. The standard errors were adjusted using Bayesian methods to enhance the stability of the statistical tests. Ultimately, differentially expressed genes (DEGs) were identified by setting thresholds (P-value < 0.05 and |logFC| > 0). These DEGs were further classified into upregulated and downregulated genes based on their logFC values. To visually illustrate the significance and direction of the differentially expressed genes, we employed the 'ggscatter' function from the 'ggpubr' package to generate volcano plots. In these plots, the x-axis represents the logFC, and the y-axis represents the -log10(P-value). Genes were colored according to their expression changes, indicating whether they were upregulated, downregulated, or stably expressed. Finally, the upregulated and downregulated gene sets of the differentially expressed genes were subjected to intersection analysis separately, and the results were visualized using Venn diagrams generated by the Venn diagrams website (https://bioinformatics.psb.ugent.be/webtools/Venn/) 22.

### The calculation process of CMap

DEGs were analyzed with the Kolmogorov-Smirnov test against raw data from the CMap database (https://clue.io/) to predict potential drugs for the treatment of IS. We downloaded the complete CMAP LINCS 2020 dataset from the CMap website. For data analysis, we used the cmapPy tool. Specifically, we ignored information such as drug dosage, cell lines, and duration of action to average the spectra of each compound. The enrichment score for the upregulated gene set is defined as 16:



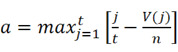



Here, *t* is the number of genes in the query gene set, *n* is the total number of genes associated with a single compound, and *V(j)* is the rank of a specific gene in the ranked list. Conversely, the enrichment score for the downregulated gene set is given by 16:



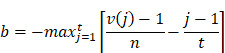



b is a metric that evaluates a compound's effect on gene downregulation by analyzing the positional distribution of downregulated genes in the expression profile, indicating the compound's regulatory potential. The overall score is defined as 16:



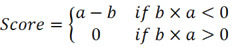



The overall connectivity score is a rating obtained by taking into account the enrichment scores, used to measure the overall similarity or dissimilarity between the input gene expression profile and the reference profile.

### Molecular fingerprints

Avalon Fingerprint enumerates feature types of the molecule subgraph and certain paths using a molecular enumerator, which contains the information of atom, bond, ring, and feature pairs 23. The molecules were coded to bit implicitly when enumerated using a hash function. Extended Connectivity Fingerprints with radius = 2 (ECFP4) are formed by substructure information of the molecular structure within a specific radius 23. The substructure information mainly contains atomic charge, non-hydrogen bond, the number of heavy atomic connections, and absolute charge. Functional-Class Fingerprints with radius = 2 (FCFP4) are similar to ECFP4. This calculation process is identical to the ECFP4 except starting with a pharmacophore set of initial atom identifiers. Molecular access system keys (MACCS) pre-defined 166 substructure fragments known as MDL Public keys 24, which consist of a string of binary digits that describe the structural characteristics of molecules. RDKit topological fingerprint was inspired by Daylight Fingerprint (https://www.daylight.com/) 25, which calculates atomic type, aromaticity, and bond type information of the substructure between minPath and maxPath, which was encoded as numeric identifiers using a hash function. Physicochemical structure descriptor (Chempy) comprises physicochemical properties and fraction of a substructure information. The former with a dimension of 123 included quantitative estimate of drug-likeness (QED), molecular weight (MolWt), topological polar surface area (TPSA), etc properties, while the latter with a dimension of 85 mainly embodied 'fr_benzene', 'fr_sulfide', 'fr_urea', etc substructure information 26. The above-mentioned six molecule fingerprints were calculated by RDKit (http://www.rdkit.org/).

### Machine learning methods

SVM is found in a hyperplane in a multidimensional space that maximizes the margin between the support vectors in every category, the support vectors are training samples on two-category margins. RF is an ensemble algorithm that integrates multiple decision trees based on a bagging algorithm. The final prediction result can be obtained by the average of decision tree outputs for the regression task. GBDT combined the addition model with the forward distribution algorithm. The decision tree was taken as the base function, which was constructed and finally integrated into a strong classifier after multiple iterations. For the detailed parameters description please refer to the literature 23.

### Model performance evaluation

Mean absolute error (MAE), MRE, mean squared error (MSE), root mean squared error (RMSE) and R^2^ were applied to evaluate model performance. The smaller the *MAE*, *MRE*, *MSE*, *RMSE* and the larger of *R*^2^, the better the model performance will be. Their description is as follows, N is the number of compounds, 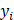
and 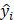
represents the actual value and the predicted value, respectively (Supplementary Table S2-S4).



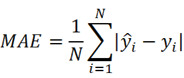





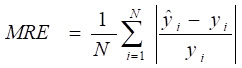





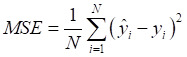





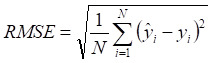





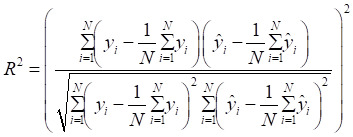



### Construction of NPAPM and compound screening

Compounds with neuroprotective properties were downloaded from the ChEMBL database (screening conditions: SH-SY5Y cell line, neuroprotection, EC_50_). 172 compounds were obtained by screening. These compounds were also converted into canonical simplified molecular input line entry system (SMILES) 27 and then transformed into Avalon, Chempy, ECFP4, FCFP4, MACCS, and RDKit molecular fingerprints, respectively. The RF, SVM, GBDT regression model were then constructed using the Python sklearn package (https://scikit-learn.org/) to predict the neuroprotective activity of launched compounds. The compounds are divided into the training set and the test set in a 4:1 ratio. The Tree-structured Parzen Estimator (TPE) optimization method was used to optimize the hyperparameters 28. RDKit molecular fingerprints were finally selected for subsequent studies. The model parameters of RDKit molecular fingerprints coupled with three models are shown in Supplementary Table S5.

### SHAP

SHAP is a method used to interpret the predictions of machine learning models. This method combines Shapley values and local interpretable model-agnostic explanations (LIME) to explain the characteristics of individual prediction instances. Shapley values are the average marginal contributions of features across all possible combinations (by taking the weighted sum of all possible feature value combinations), which can be defined by a formula. SHAP interprets the predicted value of the prediction model as the sum of the contributions of each input feature, which can be described by formula 2. The main difference between SHAP and LIME lies in the weighting calculation method between artificial samples and real instances x. LIME weights instances based on similarity, while SHAP uses the kernel function described in formula 3 for calculation 29, 30.

Formula 1:

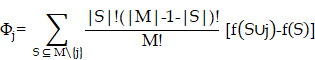



Formula 2:

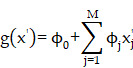



Formula 3:

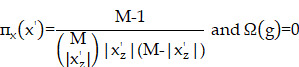



### Cell culture and cell model

SH-SY5Y cells were cultivated in DMEM enriched with 10% FBS and maintained at a temperature of 37°C within a humidified environment comprising 5% CO_2_ and 95% air. To simulate OGD/R, a well-established methodology was applied to SH-SY5Y cells. In a nutshell, SH-SY5Y cells were initially cultured under standard conditions for a 24 h period. Subsequently, they were transferred to glucose-free DMEM and exposed to ischemic conditions (95% N_2_, 5% CO_2_) at 37°C for 6 h. In the wake of the ischemic phase, the cells were incubated in conditioned DMEM (C11965500BT, Gibco, Thermo Fisher Scientific, Inc.), with or without SUL (0.1, 1, 10 μM), for a duration of 24 h.

### Cell viability assay

The related drugs were initially dissolved in dimethyl sulfoxide (DMSO, 20688, Thermo Fisher Scientific, Inc.) and subsequently diluted to a final concentration of 10 μM using DMEM supplemented with 10% FBS. Cell viability assay was performed as in the previous study 23. In brief, before concluding the treatment, MTT was added into each well, reaching a final concentration of 5 mg/mL, and allowed to incubate for 4 h. After this incubation period, the medium was aspirated, and DMSO was added into each well. The absorbance of each well was quantified using a microplate reader (Synergy H1, BioTek Instruments, Inc.), measuring the optical density at 490 nm using an enzyme meter.

### MCAO/R model establishment

Adult male Sprague-Dawley rats weighing 220 - 250 g were obtained from Liaoning Changsheng Biotechnology Co., Ltd. The experiment adhered to the guidelines outlined by the Experimental Animal Administration of the State Science and Technology Commission of the People's Republic of China, and all animal-related procedures were approved by the Animal and Medical Ethics Committee of Northeastern University. Before the surgery, rats were randomly assigned to different groups: the sham operation group, the sham operation plus 50 mg/kg SUL group (referred to as the sham + H-SUL group), the MCAO/R group, the multiple-dose SUL groups (12.5 mg/kg: L-SUL, 25 mg/kg: M-SUL, and 50 mg/kg: H-SUL), and the N-Butylphthalide (NBP) group. The sham + H-SUL group underwent a sham operation simultaneously with the sham operation group, while the MCAO/R group, the multiple-dose SUL groups (12.5 mg/kg: L-SUL, 25 mg/kg: M-SUL, and 50 mg/kg: H-SUL), and the NBP group underwent an MCAO/R operation. The experimental procedure was conducted following the principles of double-blind methodology. The experimental procedure commenced by administering anesthesia to the rats using 2.5% tribromoethanol. To induce the desired conditions, a silicone rubber-coated monofilament was cautiously inserted through a small incision into the common carotid artery and then carefully advanced into the internal carotid artery. Throughout the entire procedure, the animals were vigilantly maintained under anesthesia, which was sustained for a 2 h occlusion period. The reperfusion phase was initiated upon the removal of the monofilament. In parallel, the sham group underwent an analogous procedure as outlined above, except for inserting the monofilament 31, 32. Reperfusion was initiated 2 h after inducing cerebral ischemia in rats. SUL was administered orally at various doses of 12.5, 25, and 50 mg/kg once daily from day 1 to day 7. In contrast, rats in the sham and model groups were given saline via the same route of administration for comparative purposes. Rats in the sham + H-SUL group were administered SUL at a dose of 50 mg/kg using the same administration method. The animal usage details can be found in Supplementary Table S6.

### Neurological deficit evaluation and behavioral analysis

The assessment of neurological deficits was conducted using the mNSS before and during 1, 3, and 7 days after MCAO/R 33. The mNSS scoring system integrates multiple behavioral tests items and quantifies the neurological status of animals through detailed grading criteria. Specifically, the mNSS score encompasses various aspects, including reflex activity, motor function, balance ability, sensory function, and level of consciousness. Each item is scored on a scale from 0 to 4, with 0 typically indicating normal function and 4 indicating severe functional deficits. The total score, obtained by summing the scores of all items, reflects the severity of neurological deficits, with higher total scores indicating more severe deficits. This scoring method provides a comprehensive reflection of the animals' neurological status following stroke, offering crucial behavioral evidence for studying stroke pathogenesis and evaluating the effects of potential therapeutic interventions (Supplementary Table S7).

Specifically, a 7-day training program was implemented before inducing MCAO/R in rats, with three training sessions per day, each lasting 120 min and separated by a 120 min rest period. The rats' motor abilities were assessed by gradually increasing the speed of the rotarod from 4 to 40 revolutions per minute over 300 s. The duration before falling was recorded on days 1, 3, and 7 after MCAO/R 34, 35.

### MRI

We utilized the M5 Compact MRI System (Aspect Imaging Ltd., Israel) to perform MRI, specifically employing a rat head coil with a magnetic field strength of 1 T. T2-weighted imaging was used to evaluate the infarct volume. Rats were anesthetized with 2.5% tribromoethanol, and respiration and heartbeat were continuously monitored during scanning. The imaging parameters were set as follows: field of view at 0.2 cm × 0.2 cm, number of slices at 10, slice thickness at 2 mm, slice orientation in the axial direction, and repetition time/echo time at 3000/71.15 ms. T2-weighted images were collected *in vivo* on days 1, 3, and 7 after MCAO/R. The total scanning time for each T2-weighted imaging session was approximately 15 min, encompassing the entire process from sequence setup to data acquisition and image reconstruction.

### Measurement of infarct volume and brain water content

Seven days post-MCAO/R, the rats were euthanized via lethal overdose, and their brains were promptly excised. Coronal brain sections were incubated in 2% TTC solution at 37°C for 20 min. Fixed in 4% paraformaldehyde, the liquid on the surface of the brain slices was wiped off after 24 h for photographing. Infarct volumes were quantified with ImageJ software. The brains were promptly collected and measured in terms of their wet weight. Following a 24 h drying period in an oven set at 100°C, the brains were reweighed to determine their dry weight. The brain water content was then computed using the previously described formula 31, 32.

### Immunofluorescence

The brains were initially fixed in a 4% paraformaldehyde solution. Subsequently, the tissue samples were sequentially immersed in 20% and 30% sucrose solutions up to the bottom and then sectioned using a frozen microtome (CM1950, Leica). For immunofluorescence staining, 10 μm thick sections were subjected to antigen retrieval using a 6% citrate buffer. Next, the blocking solution was carefully applied to block the samples, followed by overnight incubation with the primary antibody and subsequent incubation with the secondary antibody.

### Western blotting

Brain tissue and cells were lysed using radio immunoprecipitation assay (RIPA) buffer (P0013B, Beyotime Biotechnology) supplemented with appropriate additives. The lysate was then centrifuged, and the resulting supernatant was collected. The protein concentration in these samples was determined using a bicinchoninic acid (BCA) assay kit (P0012, Beyotime Biotechnology). Subsequently, the proteins were separated using a 12% sodium dodecyl sulfate-polyacrylamide gel electrophoresis (SDS-PAGE) system and transferred onto polyvinylidene difluoride (PVDF) membranes. The membranes were then incubated overnight at 4°C with primary antibodies. Detected under luminometer after incubation with secondary antibody. Primary antibodies were used as follows: anti-B-cell lymphoma 2 (Bcl-2, #ab241548; Abcam; 1:1000), anti-Bcl-2-associated X protein (Bax, #ab3191; Abcam; 1:1000), anti-beta-actin (β-actin, #ab8226; Abcam; 1:1000), phosphorylated extracellular signal-regulated kinase (p-ERK, #3192; CST; 1:1000), extracellular signal-regulated kinase (ERK, #4695; CST; 1:1000), phosphorylated c-Jun N-terminal Kinase (p-JNK, #4668; CST; 1:1000), c-Jun N-terminal Kinase (JNK, #9252; CST; 1:1000), phosphorylated p38 mitogen-activated protein kinase (p-p38, #4511; CST; 1:1000), p38 mitogen-activated protein kinase (p38, #8690; CST; 1:1000), pyruvate dehydrogenase kinase 2 (PDK2, #15647-1-AP; proteintech; 1:1000).

### RNA sequencing

The SUL high group was chosen for in-depth investigation due to its noteworthy impact on the infarct area in IS. To delve into the mechanisms underlying this effect, the ischemic hemisphere, specifically the cortex, of rat brains from the sham, MCAO/R, and SUL high groups underwent high-throughput sequencing services provided by Huada Gene Company. Subsequently, differential gene analysis and enrichment analysis were conducted using the Dr.TOM website (https://biosys.bgi.com/#/report/login).

### Drug affinity responsive target stability (DARTS)

The DARTS experiment was conducted following standard procedures 36. SH-SY5Y cells were lysed using a mammalian protein extraction reagent (MPER, 78501, Thermo Fisher Scientific, Inc.) supplemented with protease and phosphatase inhibitors. Following the preparation of cell lysates from independent biological replicates, the lysates were divided into equal volumes, with each aliquot containing 100 µg of protein. These aliquots were then incubated at 25°C for 1 h in the presence or absence of SUL. After the initial incubation, the samples were further treated by incubating them with either pronase or distilled water, as specified, for 30 min at 25°C. To stop proteolysis, a protease inhibitor solution was added to each sample. Subsequently, 5x electrophoresis sample buffer was added to the samples. The expression of target proteins was then analyzed using Western blotting to confirm the binding of the protein to SUL.

### Cellular thermal shift assay (CETSA)

The CETSA experiment was conducted following standard procedures 37. In brief, the soluble protein lysate of SH-SY5Y cells was distributed into PCR tubes and treated with SUL (10 μM) or DMSO for 2 h at room temperature before the CETSA heat pulse. Subsequently, the supernatant was exposed to various temperatures using a PCR instrument (Applied Biosystems VeritiPRO, Thermo Fisher Scientific, Inc.), followed by centrifugation at 18,000×g for 20 min at 4°C to separate the soluble fraction from the precipitates. Ultimately, the samples underwent analysis via Western blotting.

### Surface plasmon resonance (SPR)

SPR experiments were performed with a Biacore T200 SPR system (GE Healthcare). Purified PDK2 protein was immobilized on a CM5 chip. SUL was injected at a flow rate of 30 µL/min for 120 s at different concentrations (0.024-250 μM) and the dissociation time was then measured with a buffer flow for 300 s. The data were analyzed using the BIAcore T100 evaluation software. Data were analyzed using BIAcore T100 evaluation software.

### Transfection of siRNA

Knockdown of PDK2 was conducted by using specific siRNA synthesized by JTS Scientific (Wuhan, China). The sequences of siRNAs for PDK2 were as follows: sense: 5'-GACUCUUCAGCUACAUGUA-3', antisense: 5'-UACAUGUAGCUGAAGAGUC-3'. Briefly, cells were grown to 80-90% confluency at the time of transfection. siRNAs and LipoRNAi (C0535, Beyotime Biotechnology) transfection regent were mixed in DMEM (without FBS). Then, SH-SY5Y cells were incubated in these mixtures. Then, 48 h later, cells underwent OGD/R.

### Phosphorylating pyruvate dehydrogenase (PDH) activity analysis

PDH activity was assessed using a PDH activity detection kit (BC0380, Solarbio) in cell lysates. First, cell samples were collected and lysed using an appropriate lysis buffer to extract proteins. The lysate was then centrifuged to remove cell debris, and the supernatant was obtained. Protein concentration was measured. The reaction mixture was prepared by adding the cell lysate, substrate, and reaction buffer, and incubated at 37°C for a specified duration. Finally, the change in absorbance at 605 nm was measured using a microplate reader (Synergy H1, BioTek Instruments, Inc.) to calculate PDH activity.

### mtDNA copy number

Cell DNA Isolation using the TIANamp Genomic DNA Kit (GDP304-02), adhering to the manufacturer's protocol. The qRT-PCR reaction conditions and system were meticulously established following the guidelines provided by Takara's SYBR Premix Ex Taq™ kit (RR390Q). The quantification of mtDNA copy number levels was achieved by employing the mtDNA/nDNA ratio, a method derived from the relative quantitative approach as referenced.

### MitoTracker red staining

For the detection of mitochondrial morphology, MitoTracker Red CMXRos from Beyotime Biotechnology (C1035) was utilized following the instructions provided by the manufacturer. In brief, cells were incubated with MitoTracker Red CMXRos at a concentration of 5 μM, and this incubation took place at 37°C for 30 min. Subsequently, images were observed using a confocal microscope (STELLARIS 5, Leica) equipped with an oil objective.

### Measurement of adenosine triphosphate (ATP) content

ATP content measurement was performed using an Enhanced ATP assay kit (S0026, Beyotime Biotechnology). The SH-SY5Y homogenate was gathered and subsequently centrifuged at 12,000 g and 4°C for a duration of 5 min. After centrifugation, 20 μL of supernatant and 100 μL of ATP detection working solution were combined. The ATP assay working solution is prepared according to the provided instructions. Luminescence was then measured using a microplate reader (Synergy H1, BioTek Instruments, Inc.).

### JC-1 straining

The mitochondrial membrane potential change was determined using a JC-1 Assay Kit (C2003S, Beyotime Biotechnology) following the manufacturer's instructions. In brief, the cells were resuspended in 500 µL of DMEM. Then, 500 µL of JC-1 staining working solution was added to the cell suspension and incubated at 37°C for 20 min. Following the incubation, the cells were centrifuged at 600 g for 3 min. After centrifugation, the supernatant was carefully removed, and the cells were washed twice with JC-1 buffer. The cells were resuspended in JC-1 staining buffer and transferred into a 96-well plate. Fluorescence measurements were taken using a microplate reader (Synergy H1, BioTek Instruments, Inc.) with excitation light at 490 nm and emission light at 530 nm to detect the green channel, while excitation light at 525 nm and emission light at 590 nm were used to detect the red channel.

### Reactive oxygen species (ROS) content detection

To measure intracellular ROS levels, tissue sections or SH-SY5Y cells were treated with 10 μM dihydroethidium (DHE, S0063, Beyotime Biotechnology) for 30 min at 37°C. After washing the sections and cells with buffer three times, they were observed using fluorescence microscopy. For *in vitro* experiments, after 24 h of treatment with or without SUL, the cells were incubated with 10 μM 2',7'-dichlorodihydrofluorescein diacetate (DCFH-DA) Fluorescent Probe (S0033M, Beyotime Biotechnology) or 20 μM MitoSOX™ Red Mitochondrial Superoxide Indicator (M36008, Thermo Fisher Scientific, Inc.) at 37°C for 30 min. Following three washes with buffer, the fluorescence intensity of DCFH-DA was measured using an emission wavelength of 484 nm and an excitation wavelength of 525 nm. The cellular fluorescence of MitoSOX was visualized using laser confocal scanning microscopy.

### Glutathione (GSH), superoxide dismutase (SOD), malondialdehyde (MDA) and H_2_O_2_ determination assay

SH-SY5Y cells were cultured in six-well plates at a density of 3 × 10^5^ cells per well. Following treatment with OGD/R, with or without the administration of SUL, the cells were harvested, homogenized in phosphate-buffered saline (PBS), and centrifuged at 1000 g for 10 min at 4°C. The supernatants were collected for the GSH, MDA, and H_2_O_2_, as well as the activities of SOD using a microplate reader (Synergy H1, BioTek Instruments, Inc.), following the manufacturer's instructions (S0053, S0131M, S0038, S0101S, Beyotime Biotechnology).

### Statistical analysis

The data variance among multiple experimental groups was assessed using one-way analysis of variance (ANOVA) for independent samples or repeated-measures ANOVA for data with repeated measures, depending on the experimental design. For post-hoc testing, Tukey's test was conducted when the data met the assumptions of normality and homogeneity of variances, while Dunnett's T3 test was used if the data violated these assumptions. The survival analysis was performed using the Kaplan-Meier estimator, and differences among groups were compared using the log-rank test. The statistical analysis was carried out using SPSS version 25.0 software. Bar graphs were created using GraphPad Prism version 7.0. All data are presented as the mean ± SEM. A value of p < 0.05 was considered statistically significant. The statistical differences of all experiments are presented in Supplementary Table S8.

## Results

### The combination of machine learning and validation experiments to identify anti-IS neuroprotective agents

**Figure 2:**
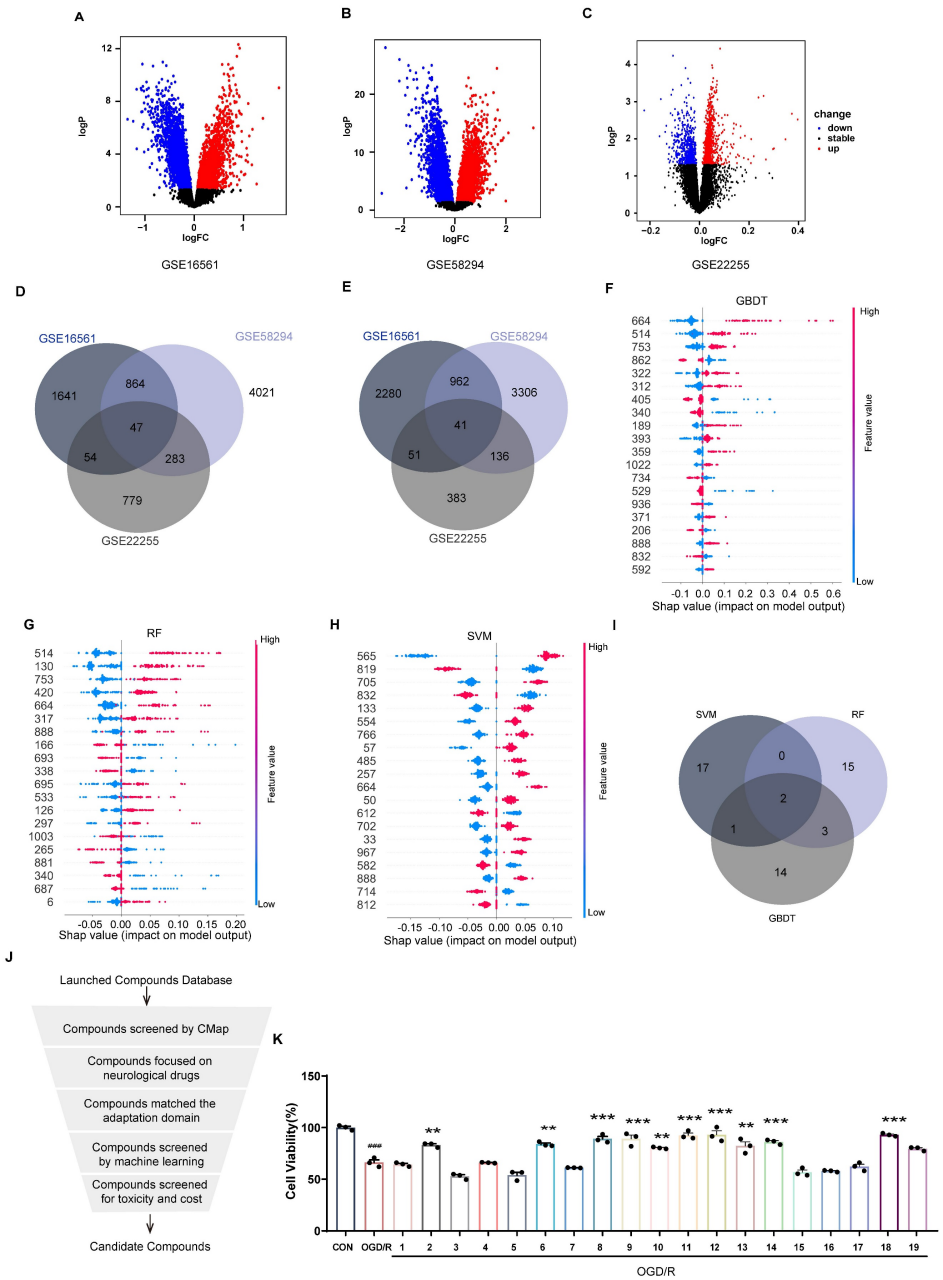

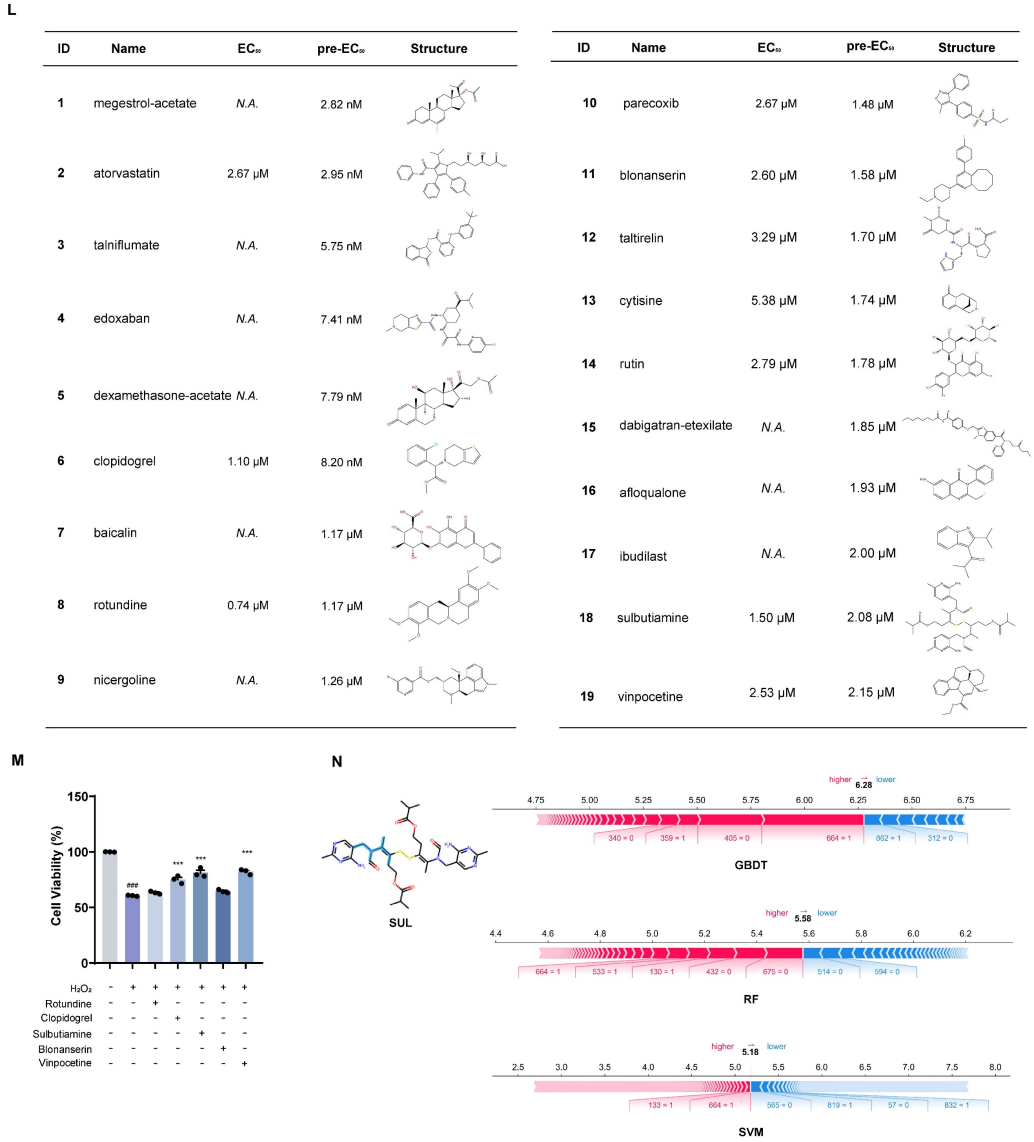
** Incorporating machine learning and validation experiments identifies anti-ischemic stroke neuroprotective agents.** (A-C) Volcano plots of the GSE16561, GSE58294, and GSE22255 differential genes. (D-E) Venn diagrams of up-and down-regulated genes for the three datasets. (F) SHAP values distribution of the Top 20 features based on GBDT models. (G) SHAP values distribution of the Top 20 features based on RF models. (H) SHAP values distribution of the Top 20 features based on SVM models. (I) Venn diagrams of the Top 20 features for the three models. (J) The flowchart for compound screening shows how to screen 19 compounds. (K) The effect of 19 compounds at 10 μM on the viability of SH-SY5Y cells was assessed using the OGD/R model (n = 3). (L) EC_50_ of compounds exhibiting neuroprotective effects in screening at 10 μM concentration. (M) The effect of the top 5 compounds at 10 μM on the viability of SH-SY5Y cells was assessed using the H_2_O_2_ model. (N) Molecular modification results and SHAP analysis for SUL (n = 3). Data are expressed as mean ± SEM. Statistics: one-way ANOVA followed by Tukey's test. ^###^*p* < 0.001 vs. control; *** *p* < 0.001, *p < 0.05 vs. OGD/R group.

Three IS-related datasets (GSE16561, GSE58294, and GSE22255) were obtained from GEO. DEGs were analyzed by LIMMA package (Figure 2A-C). The intersection of up-regulated genes among the three datasets was 47, while the intersection of down-regulated genes was 41. The intersecting genes were then submitted to the CMap database. (Figure 2D-E). The Kyoto Encyclopedia of Genes and Genomes (KEGG) enrichment analysis of crossover genes showed that DEGs were associated with various neurological diseases (Figure S1). The intersecting genes were then entered into the CMap database. To screen drugs with neuroprotective potential, a neuroprotection prediction model was constructed by three machine learning models, including RF, SVM, and GBDT, combined with six molecular fingerprints (Avalon, ECFP4, FCFP4, MACCS, RDKit, Chempy). Take the SVM model as an example, compared with the other five molecular fingerprints, the test set R^2^ of RDKit fingerprint was improved 0.1695, 0.1116, 0.0352, 0.0946, 0.2478, respectively. The comparative results, as presented in Figure S2A-C, reveal that RDKit molecular fingerprints outperformed other methods in terms of model performance. The detailed model performance metrics can be found in Supplementary Tables S2 -4, while the corresponding model hyperparameters are listed in Supplementary Table S5. Therefore, the three models were ensembled to improve the generalization ability of the neuroprotection prediction model. The performance of the ensemble model is commendable (Figure S2D-E). Then, we used the SHAP interpreter to determine which structures were identified as key structures for neuroprotection in the three models (Figure 2F-H). The results showed that features 664 and 888 were common key substructures across all three models (Figure 2I, Figure S2F). This indicates that compounds containing structures corresponding to features 664 and 888 are more likely to exhibit neuroprotective effects.

The distribution of 2419 compounds related to the disease genes was examined using the Kolmogorov-Smirnov test, leading to the screening of 1238 compounds. Considering the results of KEGG enrichment analyses, the druggability, the safety of the drugs, and their ability to cross the blood-brain barrier (BBB), we selected 287 neurological drugs for subsequent screening (Figure 2J). We then input the marketed drugs for treating neurological disorders, as screened by the CMap database, into the neuroprotection model for prediction. Out of the 93 compounds that fell within the model's applicability domain (Tanimoto similarity > 0.6), we selected the top 50 for further screening. Compounds with hepatotoxicity or high costs, as reported, were excluded 38, resulting in 19 compounds being filtered for subsequent validation.

As shown in Figure 2K, 10 compounds, including atorvastatin and clopidogrel at 10 μM, significantly increased the viability of OGD/R-induced SH-SY5Y cells. In further screening, 19 compounds were examined for neuroprotection at concentrations of 0.1, 1, 10, and 100 μM, and the EC_50_ values were calculated (Figure 2L, Figure S3). In addition, 8 of the 10 compounds tested effectively predicted EC_50_ and measured EC_50_ in the same order of magnitude, which further proved the validity of the neuroprotection prediction model. The Top 5 compounds identified were rotundine, clopidogrel, sulbutiamine, blonanserin, and vinpocetine. It is generally accepted that ischemia-reperfusion leads to the release of ROS, making neurons highly susceptible to oxidative stress injury. Therefore, the neuroprotective effects of the compounds were also evaluated in H_2_O_2_-induced oxidative stress in SH-SY5Y cells. As shown in Figure 2M, clopidogrel, sulbutiamine (SUL), and vinpocetine significantly increased the cell viability of SH-SY5Y cells exposed to H_2_O_2_. Of these, clopidogrel and vinpocetine have been reported to be clinically useful in the treatment of IS. SUL (indicated for asthma) demonstrated better effects against ischemia-reperfusion injury in both cell models and was subsequently used for further study. Next, we used the SHAP interpreter again to analyze SUL, and the results revealed the presence of the key substructure 664 and 888 in SUL. This suggests that feature 664 and 888 may be a critical substructure through which SUL exerts its neuroprotective effects (Figure 2N, Figure S2G).

### Identification of the neuroprotective effect of SUL against IS *in vitro* and *in vivo*

**Figure 3:**
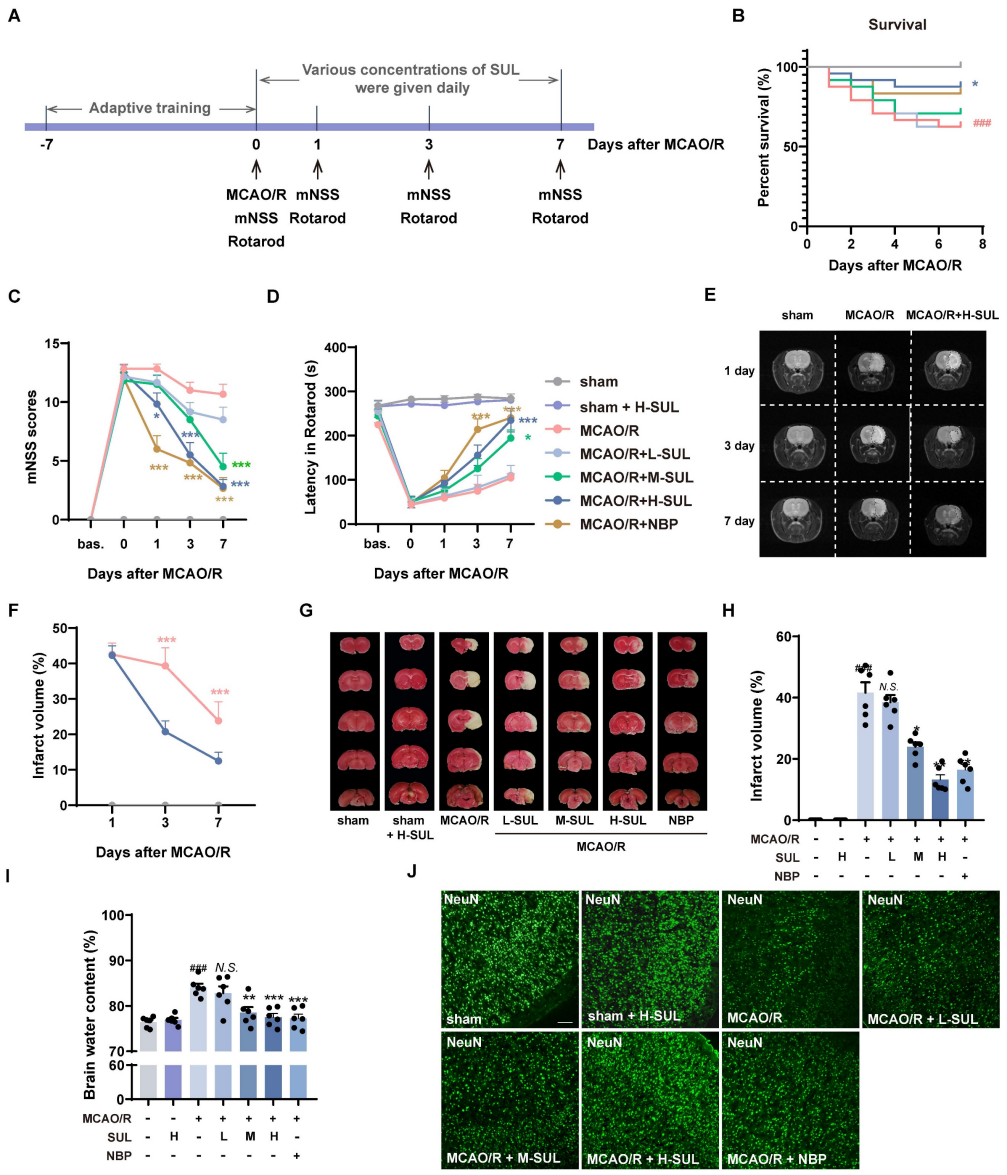

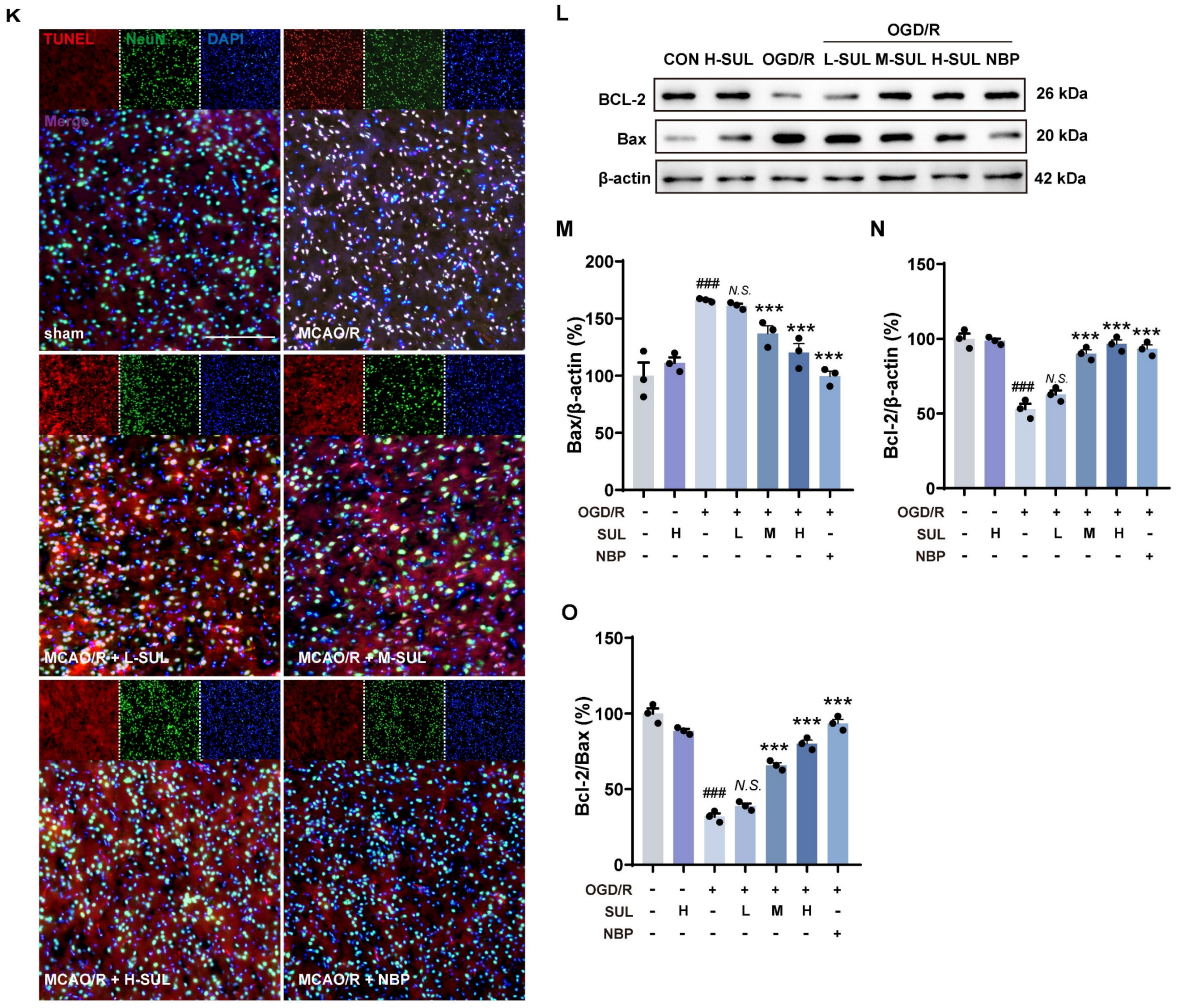
** Identification of the actual effect of SUL against ischemic stroke *in vivo* and* in vitro*.** (A) The process flow chart for MCAO/R surgery. SUL (12.5, 25, 50 mg/kg, gavage) was treated once daily from day 1 to day 7 after MCAO/R. N-butylphthalide (NBP, 80 mg/kg, gavage) was used as a positive control. (B) Effect of SUL on survival of rats within 7 days of MCAO/R (n = 24). (C-D) The mNSS and Rotarod test were evaluated before and within 7 days after MCAO/R (n = 6). (E-F) Representative MRI images showing the infarcted brain of MCAO/R rats treated with H-SUL or vehicle (n = 12). (G) Representative images showing the TTC-stained brain sections of rats treated with SUL. (H) The infarct volume was semiquantitative analyzed on day 7 after MCAO/R (n = 6). (I) Brain water content was evaluated on day 7 after MCAO/R (n = 6). (J) Immunofluorescence staining and quantification of NeuN labeled positive cells in the cerebral cortex of MCAO/R rat treated with indicated doses of SUL or vehicle (n = 6). Scale bar = 50 μm. (K) Co-localization of TUNEL- and NeuN-positive cells in the cerebral cortex of rats treated with the indicated doses of SUL or vehicle. Scale bar = 100 μm. (L-O) Immunoblot analysis of Bcl-2, Bax in SH-SY5Y treated with the indicated doses of SUL (0.1 μM: L-SUL, 1 μM: M-SUL, 10 μM: H-SUL) or vehicle (n = 3). Data are expressed as mean ± SEM. Statistics: Kaplan-Meier analysis was used for survival analysis. Repeated-measures ANOVA was applied for the data in Figures 3C-F. One-way ANOVA followed by Tukey's test was used for other comparisons. Significance levels are indicated as follows: ^###^*p* < 0.001 vs. control; *** *p* < 0.001, **p < 0.01, *p < 0.05 vs. MCAO/R group.

The MCAO/R rat model was used to evaluate the neuroprotective effects of SUL *in vivo*. The rats underwent behavioral training for 7 consecutive days before MCAO/R surgery. The related neurological function tests were performed on days 1, 3, and 7 after MCAO/R (Figure 3A).

Different dosages of SUL (12.5 mg/kg: L-SUL, 25 mg/kg: M-SUL, and 50 mg/kg: H-SUL) were administered daily during these 7 days by gavage. SUL significantly increased the survival rate of MCAO/R rats (Figure 3B). The modified Neurologic Severity Score (mNSS) significantly increased in MCAO/R rats, which was downregulated by the treatment with SUL in a dose-dependent manner (Figure 3C). In addition, compared with sham-operated rats, MCAO/R rats had a shorter rotation duration. SUL significantly increased the rotation duration in MCAO/R rats (Figure 3D). These findings demonstrate, for the first time, that SUL markedly ameliorates neurological impairments in rats subjected to MCAO/R.

To evaluate the dynamic pathophysiological response of SUL in MCAO/R-induced IS, we utilized T2-weighted magnetic resonance imaging (MRI) sequences. On days 1, 3, and 7 after MCAO/R, high signal intensities were observed in the ischemic lesions in the T2-weighted MRIs. However, treatment with SUL was found to attenuate the increase in lesion size (Figure 3E-F, Figure S4A). TTC staining showed that the cerebral infarct area significantly increased following MCAO/R, which was reduced by the treatment with SUL (Figure 3G and H). SUL markedly decreased the brain water content in MCAO/R rats (Figure 3I). The above results indicate that SUL significantly improves ischemic injury in MCAO/R rats.

Acute ischemia reperfusion (I/R) injury usually results in massive neuronal cell death, neuroinflammation, and BBB breakdown in the brain 1. Among them, neurons are the most susceptible cells in the brain to ischemic injury 39, 40. Immunostaining showed that the number of NeuN-positive cells significantly decreased in the ischemic hemisphere of MCAO/R rats, which was increased by treatment with SUL (Figure 3J, Figure S4B). Microglia and endothelial cells are components of the neurovascular unit. Our results demonstrated that MCAO/R led to a significant increase in the number of Iba-1-positive microglia, accompanied by the observation of amoeboid morphology, indicating microglial activation. Treatment with SUL effectively reduced the number of Iba-1-positive cells and improved their amoeboid cell morphology, while also partly reversing the significant decrease in the number of CD31-positive cells observed after MCAO/R (Figure S4C-E). Correlation analysis demonstrated a strong association between the effects of SUL in improving cerebral infarct volume, neurological function scores, latencies in the rotarod test, and brain water content, with the number of NeuN-positive cells (Figure S4F). Western blotting results showed that the expression level of the postsynaptic membrane protein PSD-95 was significantly reduced after MCAO/R surgery, which was reversed by SUL (Figure S4G-H).

Apoptosis is an important mode of neuronal death after ischemic injury and has been found to persist for days to weeks after an ischemic event 39. The co-localization level of terminal deoxynucleotidyl transferase-mediated dutp nick-end labeling (TUNEL) and NeuN was significantly increased after MCAO/R, and this change was attenuated by SUL (Figure 3K). Meanwhile, OGD/R resulted in a decreased expression of the anti-apoptotic protein Bcl-2 and an increase in the expression level of Bax, and both changes were attenuated by SUL (Figure 3L-O). The above results indicate that SUL inhibits neuronal apoptosis caused by I/R.

### SUL suppresses neuronal damage by suppressing the mitogen-activated protein kinase (MAPK) signaling pathway following I/R

**Figure 4:**
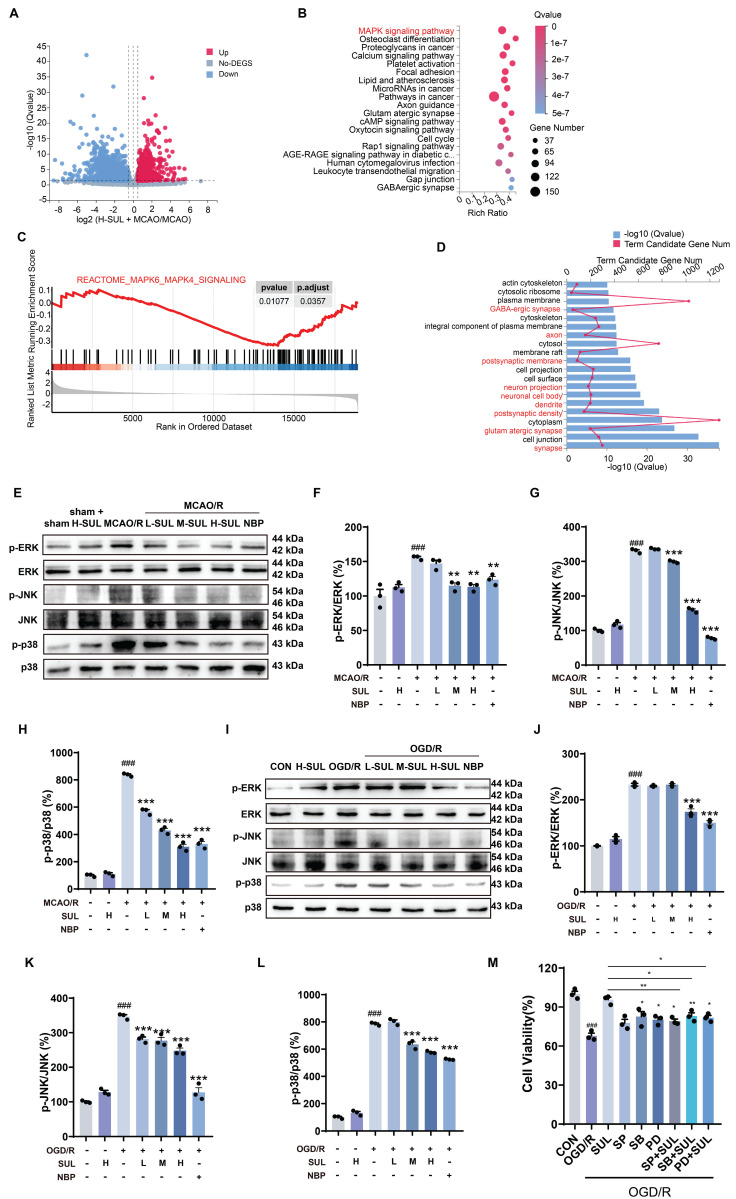
** SUL suppresses neuronal damage by suppressing the MAPK signaling pathway following I/R.** (A) Volcano maps showing the DEGs in the brain of MCAO/R rats treated with and without H-SUL (n = 3). (B) KEGG enrichment analysis showing significant enrichment of various signaling pathways in MCAO/R rats treated with and without H-SUL (n = 3). (C) GSEA analysis shows that SUL can downregulate the MAPKs signaling pathway (n = 3). (D) GO analysis showing the significant enrichment of various cellular components and cell biological processes in MCAO/R rats treated with and without H-SUL (n = 3). (E-H) Immunoblot analysis of p-ERK, ERK, p-JNK, JNK, p-p38 and p38 in the cerebral cortex of MCAO/R rats treated with the indicated doses of SUL or vehicle (n = 3). (I-L) Immunoblot analysis of p-ERK, ERK, p-JNK, JNK, p-p38 and p38 in SH-SY5Y treated with the indicated doses of SUL (0.1, 1, 10 μM) or vehicle (n = 3). (M) MTT results showed the effect of H-SUL on the activity of OGD/R-treated SH-SY5Y cells in the presence of MAPK inhibitors SB203580 (SB, 5 μM), SP600125 (SP, 10 μM), and PD98059 (PD, 20 μM) (n = 3). Data are expressed as mean ± SEM. Statistics: one-way ANOVA followed by Tukey's test. ^###^*p* < 0.001 vs. control; *** *p* < 0.001, **p < 0.01, *p < 0.05vs. MCAO/R group or OGD/R group.

The transcriptomics assay showed that a total of 4373 DEGs were identified between sham-operated and MCAO/R-operated rats. Among these DEGs, 2561 genes were found to be up-regulated, while 1812 genes were down-regulated (Figure S5A). Moreover, a total of 3340 DEGs were detected between the SUL-treated and vehicle-treated MCAO/R rats. Among these DEGs, 1396 genes were up-regulated, and 1944 genes were down-regulated (Figure 4A). KEGG enrichment analysis and GSEA analysis showed that SUL may regulate the MAPK signaling pathway (Figure 4B-C), which was consistent with those of sham-operated and MCAO/R-operated rats (Figure S5B-C). GO enrichment analysis showed that the DEGs between H-SUL- and vehicle-treated MCAO/R rats were mainly enriched in synapses, postsynaptic membranes, axons, dendrites, and other neuronal structures (Figure 4D), which was consistent with those of sham-operated and MCAO/R-operated rats (Figure S5D). These results suggest that SUL mainly regulates gene expression in neuronal structures, and its protective effect against IS may be related to the MAPK signaling pathway.

According to the transcriptomics results, the MAPK signaling pathway was further investigated. The results showed that MCAO/R notably increased the expression levels of p-ERK, p-JNK, and p-p38 in the ischemic cortex of rats, and these changes were attenuated by SUL (Figure 4E-H). Similarly, OGD/R treatment resulted in a significant up-regulation of p-ERK, p-JNK, and p-p38 proteins in SH-SY5Y cells, which were subsequently down-regulated by SUL treatment (Figure 4I-L). These findings suggest that SUL suppresses the MAPK signaling pathway following I/R.

Subsequently, we utilized MAPK inhibitors, SB203580 (SB), SP600125 (SP), and PD98059 (PD), to examine the potential involvement of MAPK in SUL-mediated alleviation of I/R-induced neuronal damage. As depicted in Figure 4M, SUL mitigated the decrease in cellular viability induced by OGD/R. PD and SB individually also enhanced cellular viability, although to a lesser extent than SUL treatment. The enhancement of cellular activity by SUL was impeded by the presence of the inhibitor. This indicates that SUL may inhibit neuronal apoptosis by suppressing the MAPK signaling pathway following I/R.

### PDK2 may be a potential target of SUL

**Figure 5:**
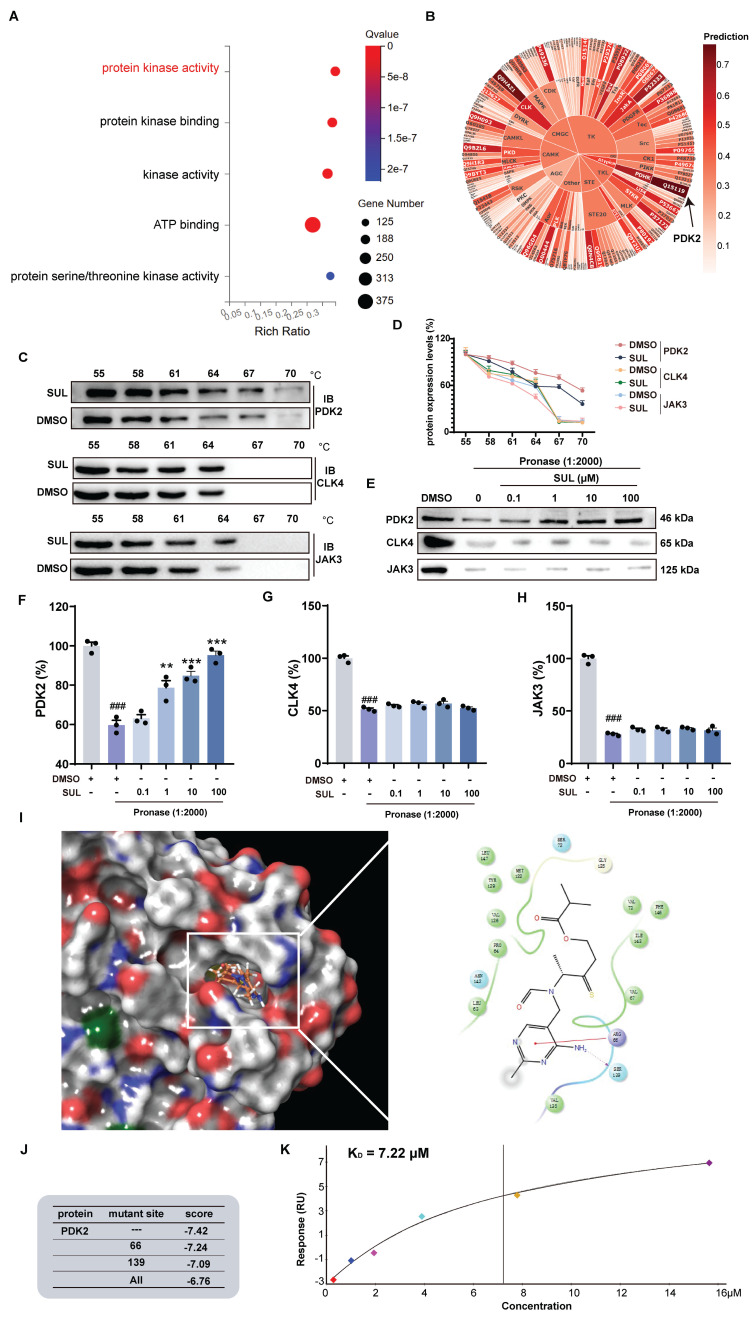
** SUL targets PDK2.** (A) GO analysis of rats treated and not treated with SUL after MCAO/R. (B) Website predicts potential targets for SUL (n = 3). (C-D) CETSA assay to detect the interaction of SUL and PDK2, CLK4 or JAK3 (n = 3). (E-H) DARTS assay to detect the interaction of SUL and PDK2, CLK4 or JAK3 (n = 3). (I) The results of molecular docking demonstrate the binding mode between SUL and PDK2. (J) The results of molecular docking show the docking scores of SUL and PDK2 after amino acid site mutations. (K) SPR analysis demonstrated the KD value of the binding between SUL and PDK2. Data are expressed as mean ± SEM. Statistics: one-way ANOVA followed by Tukey's test. ^###^*p* < 0.001 vs. DMSO; *** *p* < 0.001, **p < 0.01, *p < 0.05 vs. Pronase group.

To further investigate the potential targets of SUL and its effects, we conducted a Gene Ontology (GO) analysis of DEGs following SUL treatment. The analysis revealed that SUL may be involved in the regulation of kinase activity (Figure 5A). We then utilized the website (http://cadd.zju.edu.cn/kip/) to predict the kinases that might be inhibited by SUL 41. Among the results, PDK2 exhibited the highest predicted value (Figure 5B). To validate the predictions from the website, we employed a CETSA. SUL was analyzed with the top 3 kinases predicted by the website, and the results showed a molecular interaction between SUL and PDK2 proteins, where SUL prevented the degradation of PDK2 (Figures 5C-D). DARTS results showed that the levels of PDK2, CLK4, and JAK3 were significantly reduced by the addition of pronase, while SUL inhibited the degradation of PDK2 (Figure 5E-H).

To further analyze the binding mode of SUL and PDK2, we conducted molecular docking experiments and molecular dynamics simulation. Molecular docking results indicated that SUL may form a hydrogen bond with SER139 and a π-π interaction with ARG66 of PDK2 (Figure 5I). Molecular dynamics simulations further demonstrated that SUL can stably bind to PDK2 (Figure S5E). We performed individual and simultaneous mutations of several amino acids to verify which residues showed the strongest binding affinity with SUL. The results indicated that both single and combined mutations led to a decrease in the docking scores between SUL and PDK2. Although the decrease in docking scores is not significant, it indicates to some extent that this pocket may be an important site for the interaction between SUL and PDK2 (Figure 5J). Additionally, SPR analysis indicates that the K_D_ value between SUL and PDK2 is 7.22 μM (Figure 5K).

### SUL improves mitochondrial dysfunction by targeting PDK2

**Figure 6:**
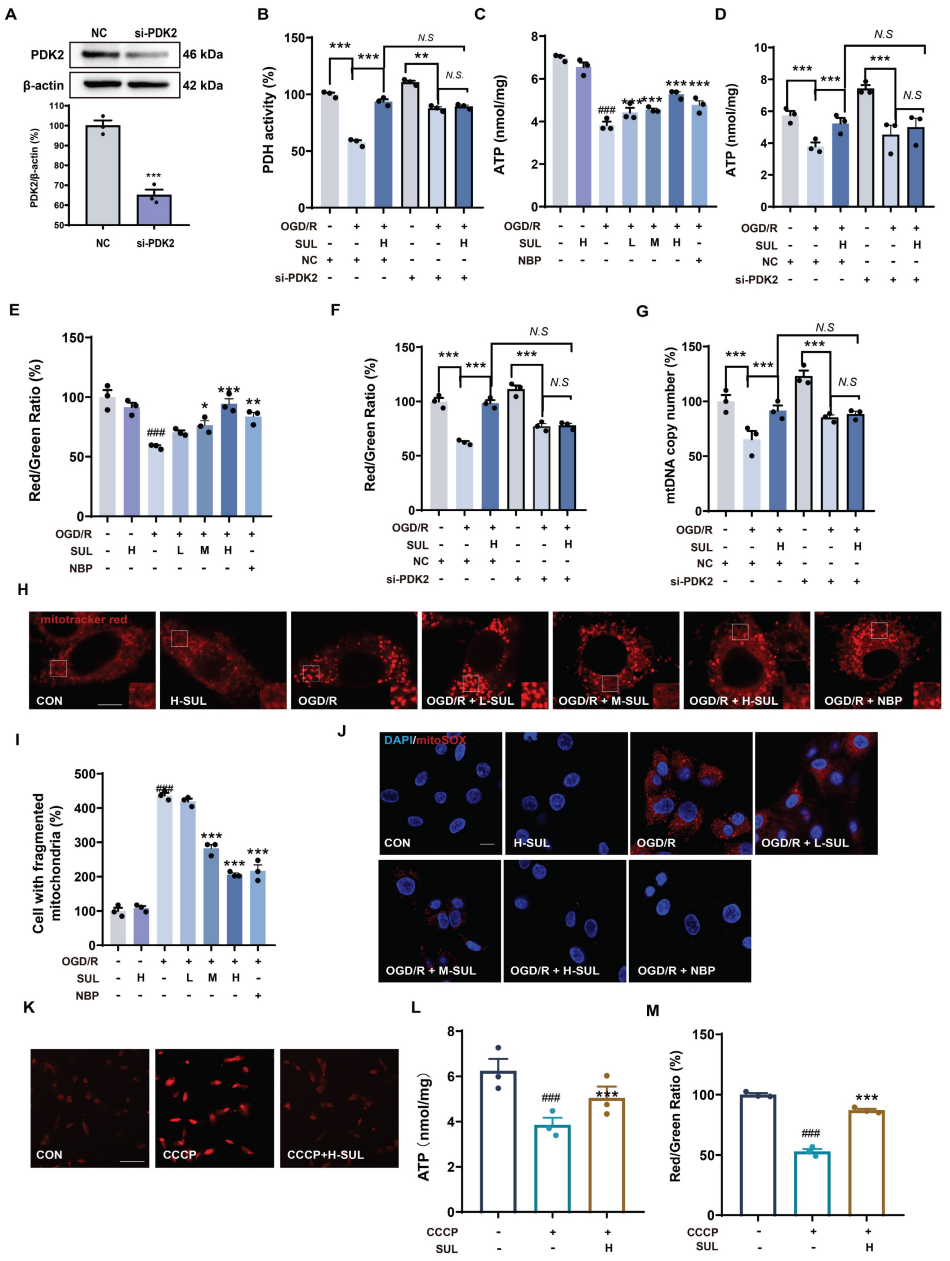
** SUL inhibits mitochondrial damage by targeting PDK2.** (A) Western blotting assay to verify the knockdown effect after transfection of siRNAs (n = 3). (B) Kit analysis showing the effect of H-SUL on PDH activity in OGD/R-treated SH-SY5Y cells after knockdown of PDK2 (n = 3). (C) ATP content after OGD/R post-SH-SY5Y (n = 3). (D) ATP content in OGD/R-treated SH-SY5Y cells after knockdown of PDK2 (n = 3). (E) JC-1 red-to-green ratio of SH-SY5Y after treatment of OGD/R (n = 3). (F) JC-1 red-to-green ratio of OGD/R-treated SH-SY5Y after knockdown of PDK2 (n = 3). (G) Kit analysis showing the effect of H-SUL on mtDNA copy number in OGD/R-treated SH-SY5Y cells after knockdown of PDK2 (n = 3). (H-I) Mitotracker red staining was performed, and fragmented mitochondria were quantified in SH-SY5Y cells after OGD/R (n = 3). Scale bar = 5 μm. (J) MitoSOX staining was applied to detect mitochondrial ROS in SH-SY5Y after treatment with OGD/R. Scale bar = 10 μm. (K) MitoSOX staining was applied to detect mitochondrial ROS in SH-SY5Y after treatment with CCCP (10 μM). Scale bar = 50 μm. (L) ATP content in SH-SY5Y cells treated with CCCP (10 μM) and then treated with H-SUL (10 μM) was detected with a commercial assay kit (n = 3). (M) JC-1 red-to-green ratio of SH-SY5Y after treatment of CCCP (10 μM) (n = 3). Data are expressed as mean ± SEM. n = 3. Statistics: one-way ANOVA followed by Tukey's test. ^###^*p* < 0.001 vs. control; *** *p* < 0.001, ** *p* < 0.01, *p < 0.05 vs. OGD/R or CCCP group.

PDK2 is an important protein that regulates key functions such as the tricarboxylic acid (TCA) cycle in mitochondria and enhances oxidative phosphorylation within the mitochondria. Studies have shown that modulating PDK2 can improve mitochondrial dysfunction 42, 43. Therefore, we further investigated the effect of SUL on mitochondrial function using PDK2 knockdown cells. SH-SY5Y cells were transfected with PDK2 small interfering RNA (siRNA) or negative control (NC) siRNA, followed by treatment with OGD/R. Transfection with PDK2 siRNA effectively decreased the expression level of PDK2 protein (Figure 6A). PDH is an important enzyme downstream of PDK2. We assessed the effect of SUL on PDH activity to reflect the impact of SUL on PDK2 activity. The results indicate that SUL can enhance PDH activity following MCAO/R (Figure S6A). As shown in Figure 6B, when comparing OGD/R + si-PDK2 with OGD/R + si-PDK2 + SUL, the effects of SUL on PDH were abolished. ATP is an indirect product of the TCA cycle. We evaluated the effect of SUL on ATP levels. After OGD/R, ATP levels were significantly downregulated, while SUL significantly upregulated ATP levels (Figure 6C). SUL also increased the ATP levels in MCAO/R rats (Figure S6B). As shown in Figure 6D, when comparing OGD/R + si-PDK2 with OGD/R + si-PDK2 + SUL, the effects of SUL on ATP were abolished. Changes in mitochondrial membrane potential can reflect the oxidative phosphorylation function within mitochondria. The results showed that after OGD/R, the JC-1 red/green ratio was significantly downregulated, while SUL significantly upregulated the JC-1 red/green ratio (Figure 6E). SUL also elevated the JC-1 red-to-green ratio in MCAO/R rats (Figure S6C). When comparing OGD/R + si-PDK2 with OGD/R + si-PDK2 + SUL, the effects of SUL on JC-1 red/green ratio were abolished (Figure 6F). When comparing OGD/R + si-PDK2 with OGD/R + si-PDK2 + SUL, the effects of SUL on the mtDNA copy number were abolished, indicating that PDK2 knockdown significantly suppressed the effect of SUL on the mtDNA copy number induced by OGD/R (Figure 6G). Confocal results further showed that after OGD/R, the mitochondrial network was fragmented, and the number of fragmented mitochondria increased. SUL treatment significantly improved these changes (Figure 6H). Furthermore, MitoSOX was used to label mitochondrial ROS. Immunofluorescence results indicated that SUL significantly reduced the increase in MitoSOX levels caused by OGD/R (Figure 6I). Next, we investigated the effect of SUL on mitochondrial oxidative phosphorylation using the uncoupler carbonyl cyanide 3-chlorophenylhydrazone (CCCP). The results showed that H-SUL downregulated the intracellular MitoSOX red fluorescence intensity induced by CCCP (Figure 6J), and upregulated the intracellular ATP levels and JC-1 red/green ratio induced by CCCP (Figure 6K-M). These findings suggest that SUL can improve mitochondrial dysfunction by targeting PDK2.

**Figure 7:**
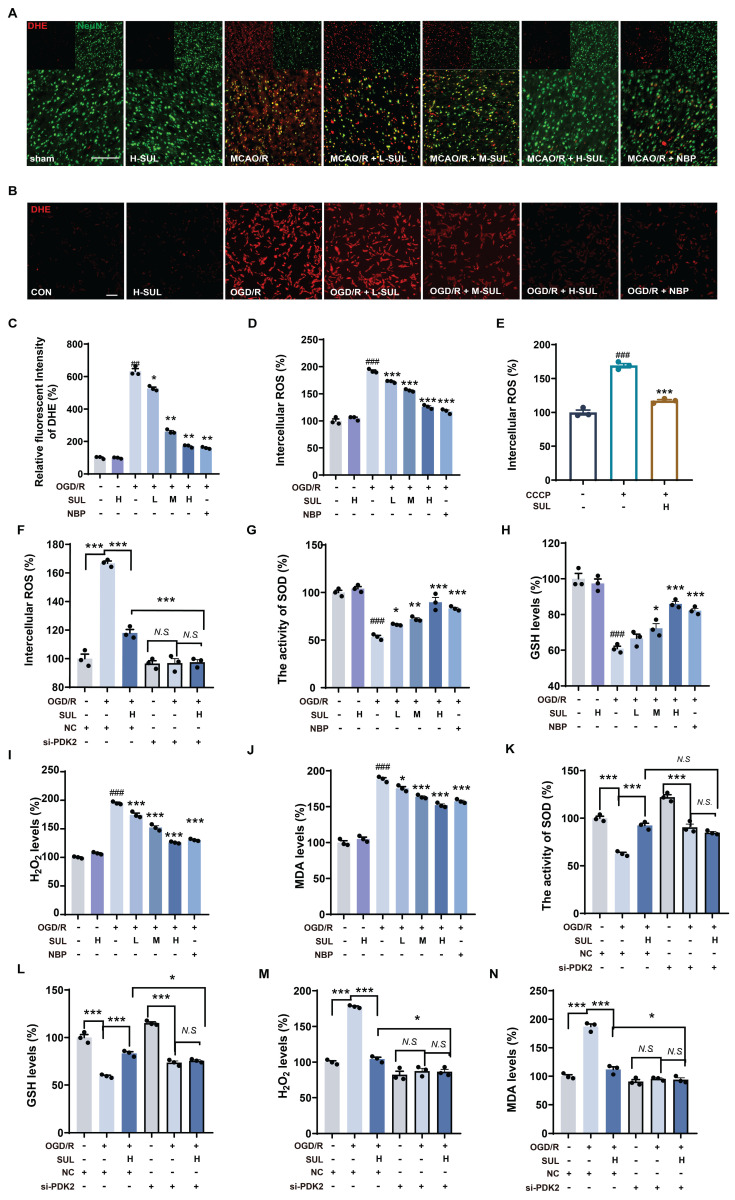
** SUL improvement of oxidative damage by targeting PDK2.** (A) Co-localization of DHE- and NeuN-positive cells in the cerebral cortex of rats treated with the indicated doses of SUL or vehicle. Scale bar = 100 μm. (B-C) DHE staining was conducted, and the intensity of DHE fluorescence was quantified in SH-SY5Y cells (n = 3). Scale bar = 50 μm. (D) The intracellular ROS levels were measured using a DCFH-DA fluorescent probe in SH-SY5Y cells (n = 3). (E) The levels of intracellular ROS in SH-SY5Y cells treated with CCCP (10 μM) and then treated with H-SUL (10 μM) were detected with DCFH-DA probe (n = 3). (F) The intracellular ROS levels were measured using a DCFH-DA fluorescent probe in SH-SY5Y cells after knockdown of PDK2 (n = 3). (G-J) The activity of SOD and the levels of GSH, H_2_O_2_, and MDA were measured using commercial assay kits (n = 3). (K-N) The activity of SOD and the levels of GSH, H_2_O_2_, and MDA were measured after knockdown of PDK2 (n = 3). Data are expressed as mean ± SEM. Statistics: one-way ANOVA followed by Tukey's test. ^###^*p* < 0.001 vs. control; *** *p* < 0.001, *p < 0.05 vs. OGD/R group.

Mitochondrial dysfunction leads to the accumulation of large amounts of ROS, and additionally, the accumulation of ROS is a key factor in neuronal damage. Immunofluorescence results showed that MCAO/R significantly upregulated the co-localization levels of NeuN and DHE, while SUL treatment reduced this change (Figure 7A). Furthermore, after OGD/R, the DHE intensity and intracellular ROS levels in SH-SY5Y cells significantly increased, and SUL concentration-dependently reduced these changes (Figure 7B-D). SUL reduced the release of ROS induced by the mitochondrial damage inducer CCCP (Figure 7E). After knocking down PDK2, SUL cannot further reduce ROS levels (Figure 7F). Mitochondrial dysfunction exacerbates oxidative stress, affecting the cell's antioxidant defense ability. Regulation of mitochondrial function can influence ROS production, leading to the accumulation of H_2_O_2_. Meanwhile, the increased level of lipid peroxidation product MDA reflects the oxidative stress state within the cells. SOD plays a key role in the antioxidant defense mechanism by converting superoxide anions (O_2_⁻) into H_2_O_2_, while GSH mitigates oxidative damage to cells by reducing H_2_O_2_ and participates in the elimination of other reactive oxygen species. Therefore, we measured H_2_O_2_ levels, SOD activity, GSH levels, and MDA concentrations to assess the effect of SUL on oxidative stress. The results showed that SUL enhanced SOD activity and GSH levels, while reducing H_2_O_2_ and MDA levels (Figure 7G-J, Figure S6D-G). Knockdown of PDK2 significantly weakened the regulation of H_2_O_2_ levels, SOD activity, GSH levels, and MDA concentrations by SUL (Figure 7K-N).

### SUL reduces ROS levels by targeting PDK2 to inhibit the MAPK signaling pathway and neuronal apoptosis

**Figure 8:**
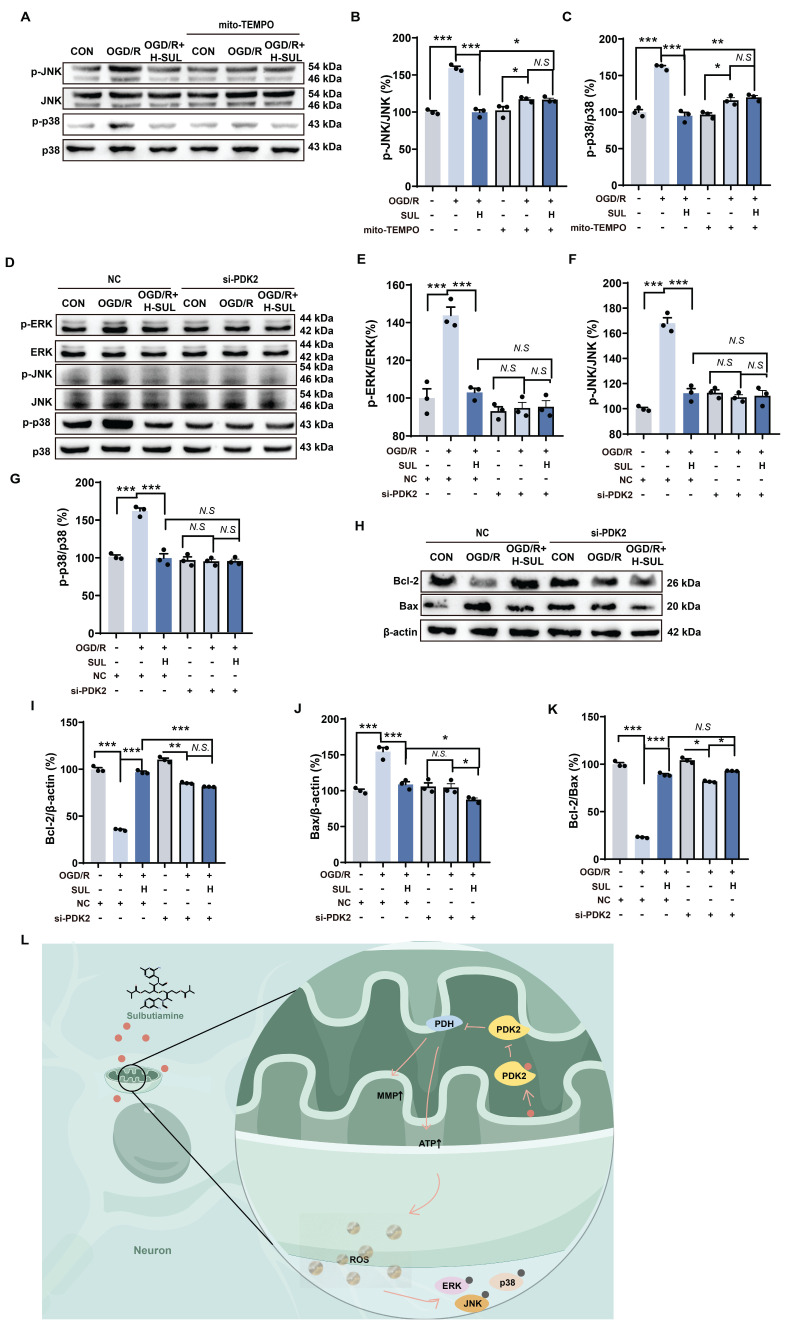
** SUL indirectly regulates MAPKs by targeting PDK2.** (A-C) Immunoblot analysis showing the expressions of p-JNK, JNK, p-p38, and p38 in OGD/R-treated SH-SY5Y cells in the presence of mito-TEMPO (10 μM) (n = 3). (D-G) Immunoblot analysis showing the expressions of p-ERK, ERK, p-JNK, JNK, p-p38, and p38 in OGD/R-treated SH-SY5Y cells after knockdown of PDK2 (n = 3). (H-K) Immunoblot analysis showing the effect of H-SUL on the expression of Bcl-2, Bax, and Bcl-2-to-Bax ratio in OGD/R-treated SH-SY5Y cells after knockdown of PDK2 (n = 3). (L) Schematic representation of SUL ameliorating mitochondrial damage by regulating PDK2. Data are expressed as mean ± SEM. Statistics: one-way ANOVA followed by Tukey's test. ^###^*p* < 0.001 vs. control; *** *p* < 0.001, *p < 0.05 vs. OGD/R group.

To further investigate the key role of mitochondrial ROS regulation in the inhibition of the MAPK signaling pathway by SUL, we introduced the mitochondrial-specific ROS inhibitor mito-TEMPO. The experimental results showed that in the presence of mito-TEMPO, the regulatory ability of SUL on p-JNK and p-p38 in cells after OGD/R treatment was significantly diminished (Figure 8A-C). Furthermore, compared to NC siRNA-transfected cells treated with SUL, cells transfected with PDK2 siRNA significantly suppressed the activation of p-ERK, p-JNK, and p-p38 induced by SUL (Figure 8D-G). More importantly, the knockdown of PDK2 significantly weakened the regulatory effect of SUL on the expression of Bax and Bcl-2 proteins induced by OGD/R (Figure 8H-K). These results indicate that SUL reduces ROS levels by targeting PDK2, thereby inhibiting the activation of the MAPK signaling pathway and mitigating neuronal apoptosis (Figure 8L).

## Discussion

In this study, we established a machine learning-based workflow, MDCDR, to identify potential neuroprotective agents from FDA approved drugs for treating IS. Our results demonstrated that SUL emerged as a promising candidate, exhibiting neuroprotective effects both *in vitro* and* in vivo*. The neuroprotective mechanism of SUL was primarily linked to its ability to ameliorate mitochondrial dysfunction and reduce neuronal apoptosis by targeting PDK2.

The use of neuroprotective agents for the treatment of IS has been clinically validated 6-8. However, the development of these agents has been hindered by researchers' misunderstandings of the concept of neuroprotection 6-8. In recent years, with the introduction of the neurovascular unit concept, researchers have recognized that the development of neuroprotective agents should not focus solely on individual neurons but should encompass a range of cells and factors 6-8. In line with the concept of developing new neuroprotective agents, we integrated stroke transcriptome data with neuron protection-related data and proposed a novel screening strategy for neuroprotective agents for the first time. This method integrates various factors by combining machine learning with transcriptome data, allowing for a more comprehensive consideration of the neuroprotective landscape, which may enhance screening efficiency. Alongside this idea, machine learning has been widely applied across various fields of drug discovery, providing new strategies for drug development 16, 17, 23. Computational drug repurposing strategies have garnered significant attention for their potential to accelerate the discovery of drugs for neurological disorders 13, 44. Combining machine learning algorithms with public transcriptome databases, such as CMap, offers a promising avenue for identifying novel therapeutic agents 13, 45, 46. To assess the advantages of integrating CMap and machine learning techniques in developing novel neuroprotectants, we compared three groups: the top 93 compounds screened using CMap alone, the top 93 compounds screened using the ML approach, and the top 93 compounds screened using the combined method (93 compounds were ultimately screened using the combined method). We evaluated the number of identified marketed drugs and experimentally validated compounds in each group. The results revealed that out of the 93 compounds screened solely with CMap, only 6 marketed drugs and 4 experimentally validated compounds were identified. In contrast, the machine learning screening yielded 9 marketed drugs and 10 experimentally validated compounds. The combined method ultimately identified 11 marketed drugs and 10 experimentally validated compounds. Although this advantage is not statistically significant, it still suggests that by considering both stroke transcriptome data and neuron protection-related data, we can enhance the efficiency of discovering novel neuroprotective agents. Furthermore, among the 19 compounds finalized through screening, some are clinically used for stroke treatment, including those with neuroprotective effects. This further demonstrates the validity of our strategy. Additionally, we applied the SHAP interpreter to explore important substructures related to neuroprotection. The results indicate that structure 664 may be a crucial substructure for neuroprotective effects, and SUL, which contains this structure, also demonstrates significant neuroprotective activity. At present, the amount of modeling data in the study is 172. With the in-depth study on stroke research, the amount of sample data available is gradually increasing. Active learning can be adopted for further modeling, so as to improve the generalization ability of the model and broaden the application area of the model. Additionally, the current study focuses on FDA approved drugs with central nervous system penetration potential, prioritizing translational feasibility, which indeed limits the universality of the workflow.

In the future, we plan to expand the model to a broader range of drug libraries, including non-neurological drugs. During the expansion, we will employ data standardization and feature engineering to ensure the compatibility of the newly incorporated drug data with the existing model. Meanwhile, we will integrate emerging data sources such as single-cell transcriptomics to identify truly novel drug repurposing candidates. To ensure the validity of the improved model, we will conduct large-scale external validation and comparative analysis with other independent research datasets. Moreover, we will collaborate with clinicians and drug development experts to evaluate and optimize the model from a clinical application perspective. These series of improvement measures will further enhance the universality and innovativeness of the workflow.

Among the 19 compounds screened, SUL was identified as the most significant neuroprotective agent. Although some of the other 19 compounds have been reported to have neuroprotective effects, they pose various risks compared to SUL. For example, clopidogrel is used for secondary prevention of stroke (as an antiplatelet therapy), but it lacks direct neuroprotective effects and is associated with bleeding risks 47. Atorvastatin has been found to increase the risk of diabetes, and statins are prone to causing bleeding risks 48. Moreover, compounds such as baicalin, rotundine, and nicergoline have been studied, but our research system did not demonstrate their significant neuroprotective effects, or they have issues such as low bioavailability and cytotoxicity at high concentrations 49, 50. SUL is a lipophilic synthetic compound formed by the linkage of two thiamine (vitamin B1) molecules via a disulfide bond, which enhances its permeability across the blood-brain barrier compared to thiamine 51. Consequently, SUL can increase the levels of thiamine and thiamine phosphate in the brain 51-53. Research indicates that SUL may have various potential effects on central nervous system diseases, such as improving diabetic neuropathy, frailty syndromes, chronic weakness, and fatigue, alleviating psychogenic erectile dysfunction, enhancing long-term memory formation and motor performance, and mitigating the psycho-behavioral effects of major depression and schizophrenia, with no reports of adverse reactions to date 52, 54, 55. Our findings demonstrate that SUL significantly improved neurological function in the rat model of MCAO/R, as evidenced by increased survival rates, reduced neurological deficits, and improved performance in behavioral tests. This is particularly important considering that acute injury often leads to cell death and neuroinflammation, both of which play a crucial role in the progression of ischemic damage. The use of T2-weighted MRI indicated that SUL treatment effectively reduced lesion size, further supporting its neuroprotective effects.

In the context of ischemic injury, neurons are particularly vulnerable and often exhibit widespread pathological manifestations, including neuronal cell death, mitochondrial dysfunction, and excessive accumulation of ROS 56, 57. Overall, these findings indicate that the modulation of the MAPK pathway by SUL was associated with a mitigation of mitochondrial damage and a subsequent reduction in ROS levels. The role of the MAPK signaling pathway in neuronal death has been well established, and previous studies have shown that inhibiting the JNK and p38 signaling pathways effectively protects neurons from ischemia-induced death 58. Our results revealed that SUL treatment significantly downregulated the expression of these proteins, indicating that inhibition of this pathway is an important mechanism by which SUL exerts its protective effects. Furthermore, mitochondria serve as a major source of excessive ROS generation in the brain and play a critical role in the activation of the MAPK signaling pathway. Our study highlights that SUL can inhibit the activation of this pathway by reducing mitochondrial ROS levels, and the observed increase in superoxide dismutase activity and glutathione levels further supports SUL's role in alleviating oxidative stress. Additionally, the inhibitory effect of SUL on mitochondrial ROS is attributed to its protective effect on mitochondrial function. In summary, SUL may play a crucial neuroprotective role in ischemic injury by modulating the MAPK signaling pathway and reducing the generation of mitochondrial ROS.

PDK2 is a key protein that regulates mitochondrial processes in the TCA cycle, primarily by PDH to inhibit the conversion of pyruvate to acetyl-CoA, thereby modulating both the TCA cycle and oxidative phosphorylation 56, 59. Studies have indicated that regulating PDK2 can improve abnormal brain energy metabolism 42, making it a potential target for neuroprotection in the treatment of IS. Our results further reveal that targeting PDK2 can inhibit brain ischemic injury by improving mitochondrial dysfunction in neurons. Specifically, PDK2 functions primarily by phosphorylating the E1 subunit of the pyruvate dehydrogenase complex (PDH complex), inhibiting the conversion of pyruvate to the mitochondrial TCA cycle. Inhibiting PDK2 helps to regulate pyruvate metabolism, enhance aerobic metabolism, and ultimately promote oxidative phosphorylation 42, 56. Furthermore, complexes I and III are widely recognized as the main sites of ROS generation during oxidative phosphorylation 42, and targeting PDK2 to regulate mitochondrial oxidative phosphorylation effectively reduces the excessive generation of ROS, which subsequently influences the activation of the MAPK signaling pathway. Meanwhile, our results reveal that silencing PDK2 can significantly inhibit the release of ROS, which further highlights the pivotal role of PDK2 in the treatment of ischemic stroke. Additional studies, including CETSA, DARTS, and SPR, have shown that SUL binds to PDK2, and molecular docking analyses indicate potential amino acid sites for the interaction between SUL and PDK2. Notably, we observed that the ability of SUL to restore mitochondrial function diminished after interfering with PDK2. In summary, our experimental results suggest that PDK2 is an important target for improving IS, and SUL exerts its neuroprotective effects by alleviating mitochondrial dysfunction and inhibiting apoptosis by targeting PDK2.

In summary, we developed MDCDR for screening novel neuroprotective agents by integrating transcriptomic data with neuroprotection data. This approach not only enhances the efficiency of drug screening but also surpasses the results achieved by considering a single disease factor. Furthermore, we successfully identified SUL as a potential neuroprotective agent for the treatment of IS. The anti-ischemic effects of SUL were revealed for the first time through *in vivo* and *in vitro* experiments. Its mechanism of action may involve targeting PDK2 to inhibit the MAPK signaling pathway and neuronal death, thereby restoring mitochondrial function and reducing ROS levels. Our study provides a valuable reference for the development of novel neuroprotective agents and suggests that PDK2 may be an important target for developing neuroprotective agents against IS.

## Supplementary Material

Supplementary methods, figures and tables.
