# Supplementary material for: Multi-dimensional data-driven computational drug repurposing strategy for screening novel neuroprotective agents in ischemic stroke
Source: Theranostics. 2025 Jun 23;15(15):7653–76. doi: 10.7150/thno.112608 (PMC12316036; doi:10.7150/thno.112608)
Supplement: Supplementary file 1 — Supplementary methods, figures and tables. [file thnov15p7653s1.pdf]

Supplementary Materials

Multi-dimensional data-driven computational drug repurposing strategy for screening novel neuroprotective agents in ischemic stroke

Contents:

Extend Method. Enrichment analysis
Extend Method. Molecular docking
Extend Method. Molecular Dynamics (MD) Simulation
Extend Method. Phosphorylating pyruvate dehydrogenase (PDH) activity analysis
Extend Method. Measurement of adenosine triphosphate (ATP) content
Extend Method. Glutathione (GSH), superoxide dismutase (SOD), malondialdehyde (MDA) and H_2O_2 determination assay
Extend Method. JC-1 straining
Supplementary Figure S1. Enrichment analysis of DEGs in ischemic stroke.
Supplementary Figure S2. Performance of machine learning models.
Supplementary Figure S3. Screening candidate compounds for neuroprotective effects.
Supplementary Figure S4. SUL amelioration of I/R injury is most associated with amelioration of neuronal injury.
Supplementary Figure S5. Transcriptome analysis of MCAO/R group rats and sham group rats.
Supplementary Figure S6. SUL improves mitochondrial dysfunction in MCAO/R rat.
Supplementary Table S1. Abbreviation list.
Supplementary Table S2. The model performance comparison results for different molecular fingerprints based on GBDT.
Supplementary Table S3. The model performance comparison results for different molecular fingerprints based on SVM.
Supplementary Table S4. The model performance comparison results for different molecular fingerprints based on RF.
Supplementary Table S5. The hyper-parameter for three models optimized by TPE.
Supplementary Table S6. Rat grouping and numbers.
Supplementary Table S7. Neurological function score.
Supplementary Table S8. Data statistical methods and confidence intervals.

Extend Method

Enrichment analysis

For the intersection data of differentially expressed genes (DEGs) from the three datasets, we conducted KEGG pathway enrichment analysis using the DAVID website (https://david.ncifcrf.gov/). The specific steps were as follows: the intersection data of DEGs were input into the DAVID website, "Homo sapiens" was selected as the analysis species, the KEGG pathway enrichment analysis function was chosen, and the default parameters were set.

For the KEGG, GO, and GSEA analyses of the transcriptomic data, we used the Dr.TOM website (https://biosys.bgi.com/#/report/login). The specific steps were as follows: after performing differential analysis, the groups to be analyzed were selected, and then the KEGG pathway enrichment analysis, GO functional enrichment analysis, and GSEA analysis functions were chosen.

Molecular docking

The Schrödinger software was selected for molecular docking analysis. The protein structures were obtained separately and then preprocessed using the Protein Prepare Wizard module. The Receptor Grid Generation module was utilized to generate the grid file, with the docking box set to the default size. Subsequently, the small molecule structure of SUL was obtained and preprocessed using the LigPrep module. For molecular docking, the accuracy parameter was set as XP.

Molecular Dynamics (MD) Simulation

In this study, we conducted molecular dynamics simulations using the GROMACS 2018 software with the SPC water model and AMBER force field. After separating the receptor and ligand from the complex file, we used the `pdb2gmx` tool to convert the receptor PDB file into GROMACS-compatible structure and topology files, specifying the AMBER force field and SPC water model. For the ligand, its topology file was generated via the `antechamber` program and then integrated into the receptor's topology file. Subsequently, the system was placed into a cubic box using `editconf`, and SPC water molecules were added for solvation through `genbox`. Following energy minimization, the system underwent NVT and NPT equilibration steps to reach a stable state. Finally, a 10 ns molecular dynamics simulation was performed, during which the system's conformations and energy information were recorded for subsequent analysis.

Phosphorylating pyruvate dehydrogenase (PDH) activity analysis

Approximately 0.1 g of tissue was weighed and homogenized thoroughly with 1 mL of Reagent 1 and 10 µL of Reagent 2 using a homogenizer or mortar in an ice bath. The homogenate was then centrifuged at 11,000g for 10 minutes at 4°C. The supernatant was collected and kept on ice for subsequent analysis. The activity of PDH was measured according to the instructions provided in the kit (BC0380, Solarbio).

Measurement of adenosine triphosphate (ATP) content

Tissue samples were processed at a ratio of 100–200 µL of lysis buffer

per 20 mg of tissue. After thorough homogenization, the samples were centrifuged at 12,000 g for 5 min at 4 °C. The supernatant was collected for subsequent analysis. Subsequently, the ATP content was measured precisely according to the instructions provided with the reagent kit (S0026, Beyotime Biotechnology).

JC-1 straining

The tissue was mixed with the mitochondrial isolation reagent and homogenized in an ice bath. Subsequently, the homogenate was centrifuged at 600 g for 5 min at 4°C. The supernatant was transferred to another centrifuge tube and centrifuged at 11,000 g for 10 min at 4°C (C3606, Beyotime Biotechnology). The supernatant was discarded, and the pellet was the isolated mitochondria. Subsequently, the mitochondrial membrane potential was measured according to the instructions (C2003S, Beyotime Biotechnology).

Glutathione (GSH), superoxide dismutase (SOD), malondialdehyde (MDA) and H_2O_2 determination assay

Tissue samples were first frozen rapidly with liquid nitrogen and then ground into powder. Subsequently, for every 10 mg of tissue powder, 30 µL of protein removal reagent M solution was added, followed by thorough vortexing. Then, an additional 70 µL of protein removal reagent M solution was introduced, and the mixture was homogenized thoroughly. Finally, the GSH content was measured according to the instructions provided with the

reagent kit.

Separately, an appropriate amount of tissue sample was taken and homogenized in an ice bath at a ratio of 100 μL of SOD sample preparation solution per 10 mg of tissue. The homogenate was then centrifuged at approximately 12,000 g for 3 – 5 min at 4°C, and the supernatant was collected as the sample for testing. The SOD activity was measured in accordance with the instructions provided with the reagent kit.

For the measurement of hydrogen peroxide content, tissue samples were homogenized at a ratio of 100 – 200 μL of lysis buffer per 5 – 10 mg of tissue. The homogenate was centrifuged at approximately 12,000 g for 3 – 5 min at 4°C, and the supernatant was collected for subsequent analysis, following the instructions provided with the reagent kit.

When determining the MDA content, the tissue samples were homogenized with lysis buffer first, and then the protein concentration was measured using the BCA method. Finally, the MDA content was measured according to the instructions provided with the reagent kit (S0053, S0131M, S0038, S0101S, Beyotime Biotechnology).

Legends

Supplementary Figure S1. Enrichment analysis of DEGs in ischemic stroke.

This figure illustrates the KEGG pathway analysis of genes that are co-upregulated and co-downregulated when comparing healthy individuals to ischemic stroke patients. The analysis highlights key pathways involved, offering insights into the biological processes affected by ischemic stroke.

Supplementary Figure S2. Performance of machine learning models.

(A) The performance of the GBDT model was evaluated using metrics such as MRE, MAE, MSE, RMSE, and R^2. (B) Evaluation of the SVM model was conducted with metrics including MRE, MAE, MSE, RMSE, and R^2. (C) The RF model performance was assessed using metrics like MRE, MAE, MSE, RMSE, and R^2. (D) Training curve of the Ensemble model. (E) The testing curve of the Ensemble model. (F) The important sub-structure 664 and 888. (G) The important sub-structure 888 in SUL.

Supplementary Figure S3. Screening candidate compounds for neuroprotective effects.

The effects of the following compounds on the viability of OGD/R-treated SH-SY5Y cells were detected using an MTT assay: (A) megestrol acetate (0.1, 1, 10, and 100 μM), (B) atorvastatin (0.1, 1, 10, and 100 μM), (C) talniflumate (0.1, 1, 10, and 100 μM), (D) edoxaban (0.1, 1, 10, and 100 μM), (E) dexamethasone acetate (0.1, 1, 10, and 100 μM), (F) clopidogrel (0.1, 1, 10,

and 100 μM), (G) baicalin (0.1, 1, 10, and 100 μM), (H) rotundine (0.1, 1, 10, and 100 μM), (I) nicergoline (0.1, 1, 10, and 100 μM), (J) parecoxib (0.1, 1, 10, and 100 μM), (K) blonanserin (0.1, 1, 10, and 100 μM), (L) taltirelin (0.1, 1, 10, and 100 μM), (M) cytisine (0.1, 1, 10, and 100 μM), (N) rutin (0.1, 1, 10, and 100 μM), (O) dabigatran etexilate (0.1, 1, 10, and 100 μM), (P) afloqualone (0.1, 1, 10, and 100 μM), (Q) ibudilast (0.1, 1, 10, and 100 μM), (R) sulbutiamine (0.1, 1, 10, and 100 μM), and (S) vinpocetine (0.1, 1, 10, and 100 μM). Data are expressed as mean ± SEM. Statistics: one-way ANOVA followed by Tukey's test. $^{###}p < 0.001$ vs. control; *** $p < 0.001$, $^{**}p < 0.01$, $^{*}p < 0.05$ vs. OGD/R group.

Supplementary Figure S4. SUL amelioration of I/R injury is most associated with amelioration of neuronal injury.

(A) Representative MRI images showing the infarcted brain of MCAO/R rats treated with H-SUL or vehicle (n = 12). (B) Quantification of NeuN-labeled positive cells in the cerebral cortex of MCAO/R rats treated with indicated doses of SUL or vehicle (n = 6). (C-E) Immunofluorescence staining and quantification of Iba-1 and CD31-labeled positive cells in the cerebral cortex of MCAO/R rats treated with indicated doses of SUL or vehicle. Scale bar = 50 μm (n = 6). (F) Heatmap of absolute values of NeuN, Iba-1, and CD31 correlation with infarct volume, mNSS scores, Latency in rotarod, and brain water content. (G-H) Immunoblot analysis of PSD-95 in the cerebral cortex of MCAO/R rats treated with the indicated doses of SUL or vehicle (n = 3). Data

are expressed as mean ± SEM. Statistics: one-way ANOVA followed by Tukey's test. $^{###}p < 0.001$ vs. control; *** $p < 0.001$, $^{*}p < 0.05$ vs. MCAO/R group.

Supplementary Figure S5. Transcriptome analysis of MCAO/R group rats and sham group rats.

(A) Volcano plots depicting the Differentially Expressed Genes (DEGs) in rats treated with and without MCAO/R operation. (B) KEGG analysis results illustrate the pathways enriched in rats treated with and without MCAO/R operation. (C) GO analysis results show the GO terms enriched in rats treated with and without MCAO/R operation. (D) GSEA analysis shows that the MAPK signaling pathway is upregulated in rats after MCAO/R surgery. (E) The RMSD of the PDK2-SUL complex.

Supplementary Figure S6. SUL improves mitochondrial dysfunction in MCAO/R rats.

(A) Kit analysis demonstrating the impact of SUL on PDH activity in MCAO/R rats. (B) Measurement of ATP content following MCAO/R. (C) The JC-1 red-to-green ratio in MCAO/R rats. (D-G) The activity of SOD and the levels of GSH, H_2O_2, and MDA were measured using commercial assay kits. Data are expressed as mean ± SEM. n = 3. Statistics: one-way ANOVA followed by Tukey's test. $^{###}p < 0.001$ vs. control; *** $p < 0.001$, $^{*}p < 0.05$ vs. OGD/R group.

Supplementary Figure S1

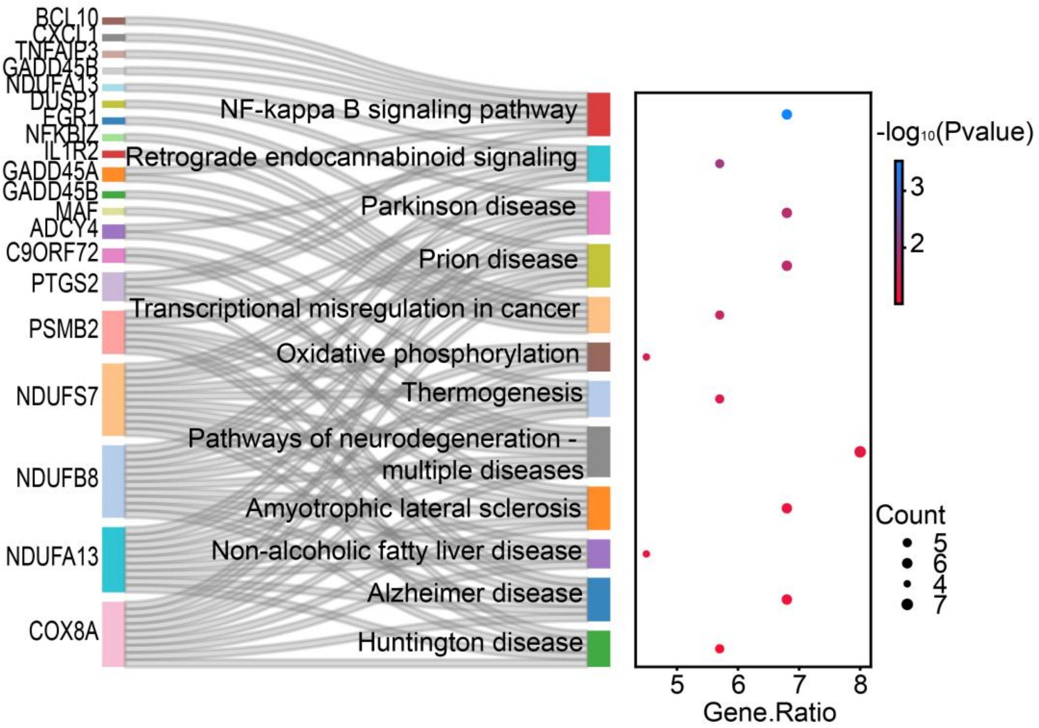

Supplementary Figure S2

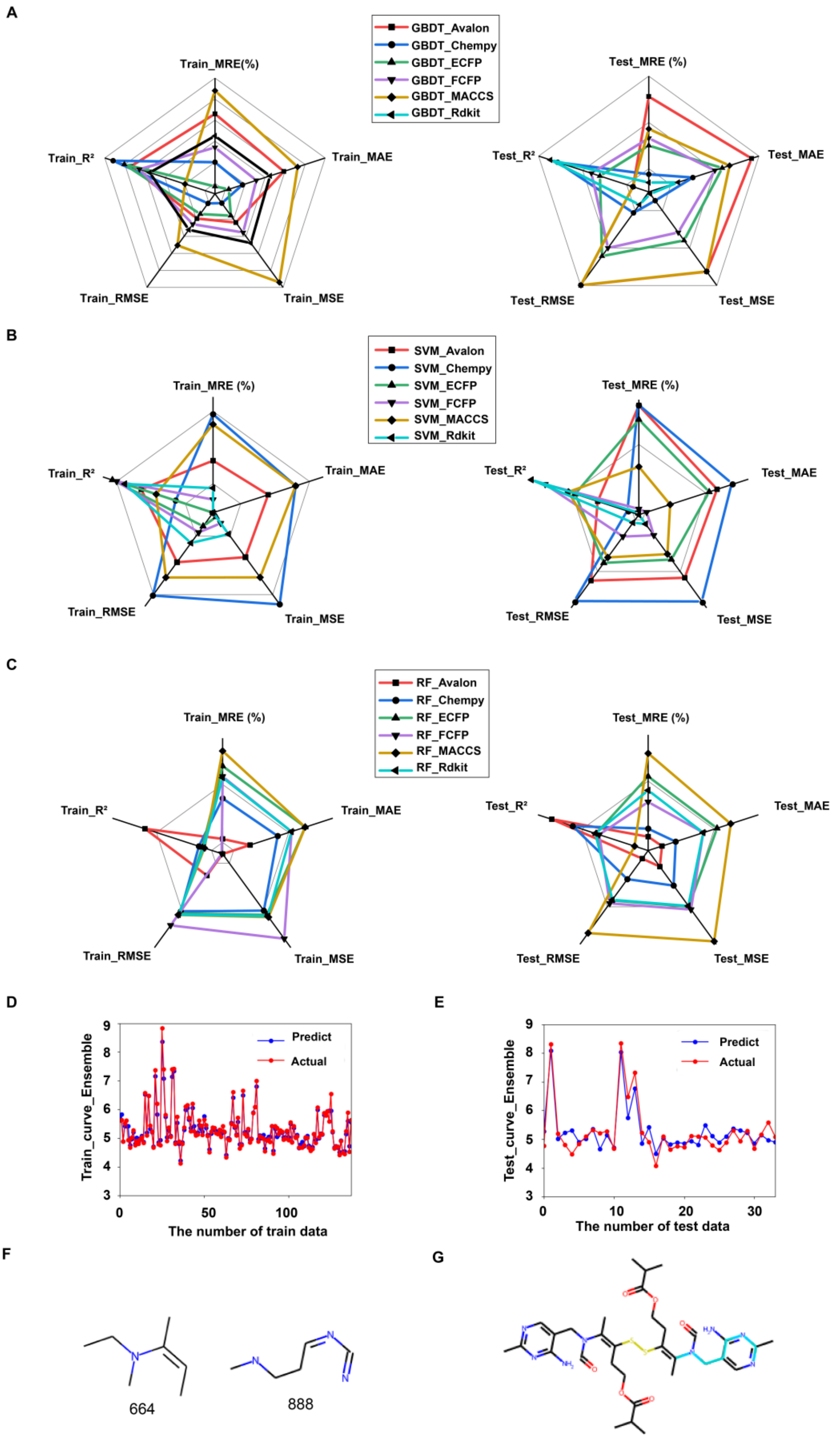

Supplementary Figure S3

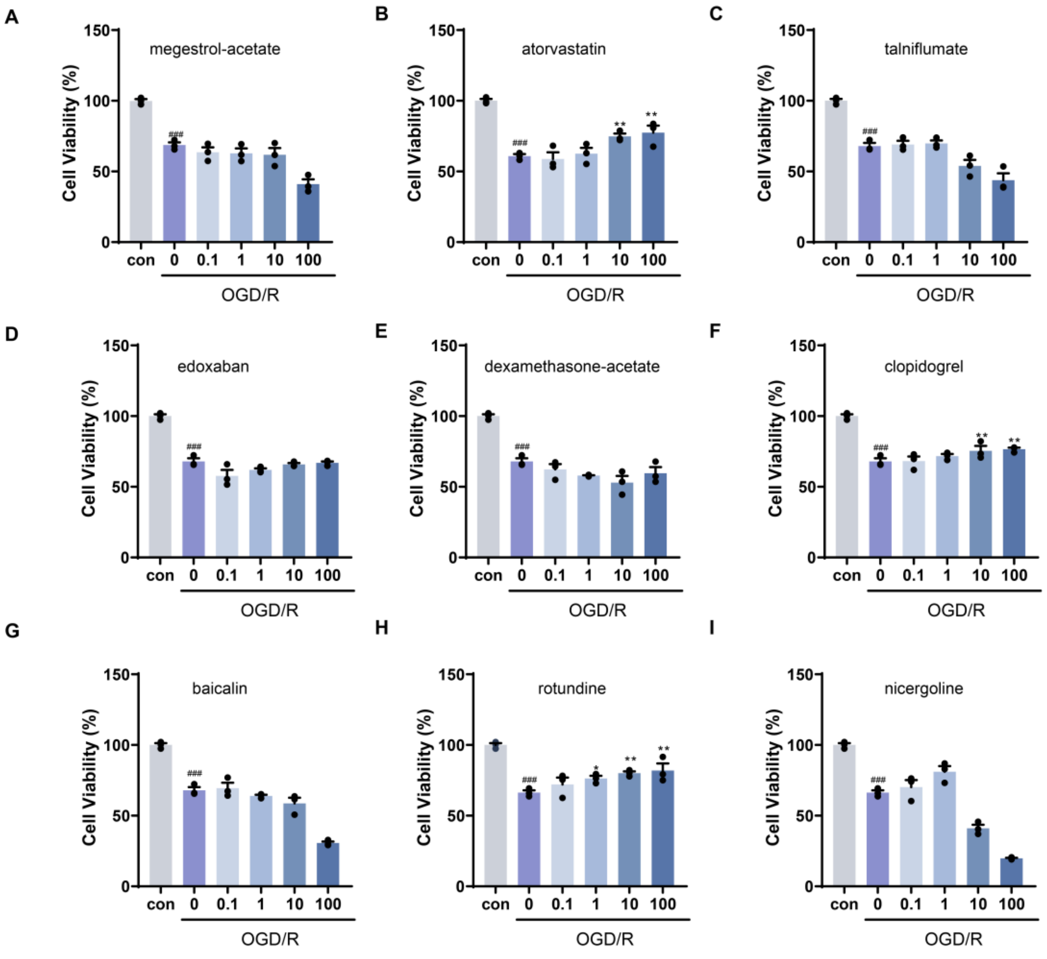

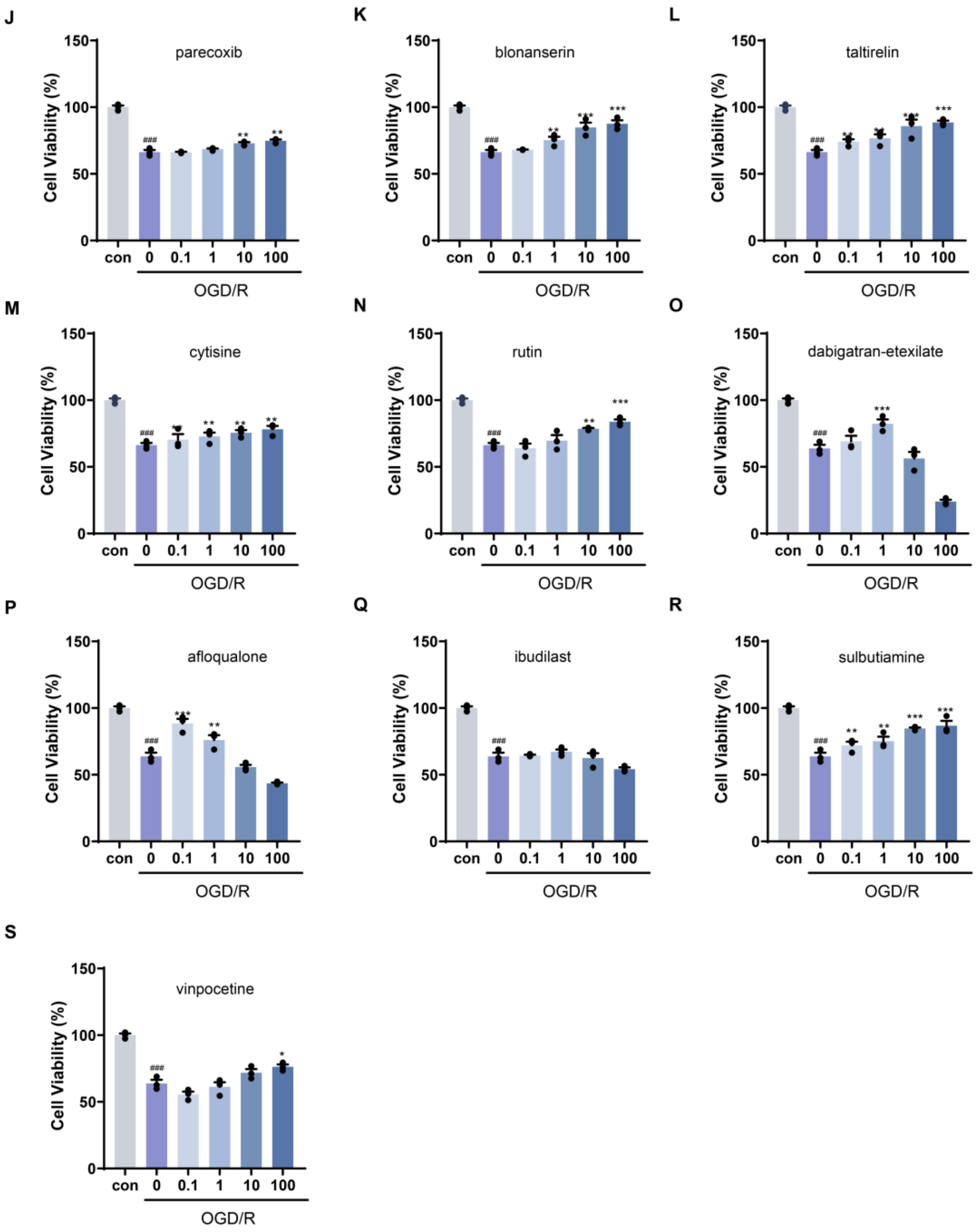
J
parecoxib
K
blonanserin
L
taltirelin
M
cytisine
N
rutin
O
dabigatran-etexilate
P
afloqualone
Q
ibudilast
R
sulbutiamine
S
vinpocetine
Cell Viability (%)
0
50
100
150
con
0
0.1
1
10
100
OGD/R
###
*
**
***

198 **Supplementary Figure S4**

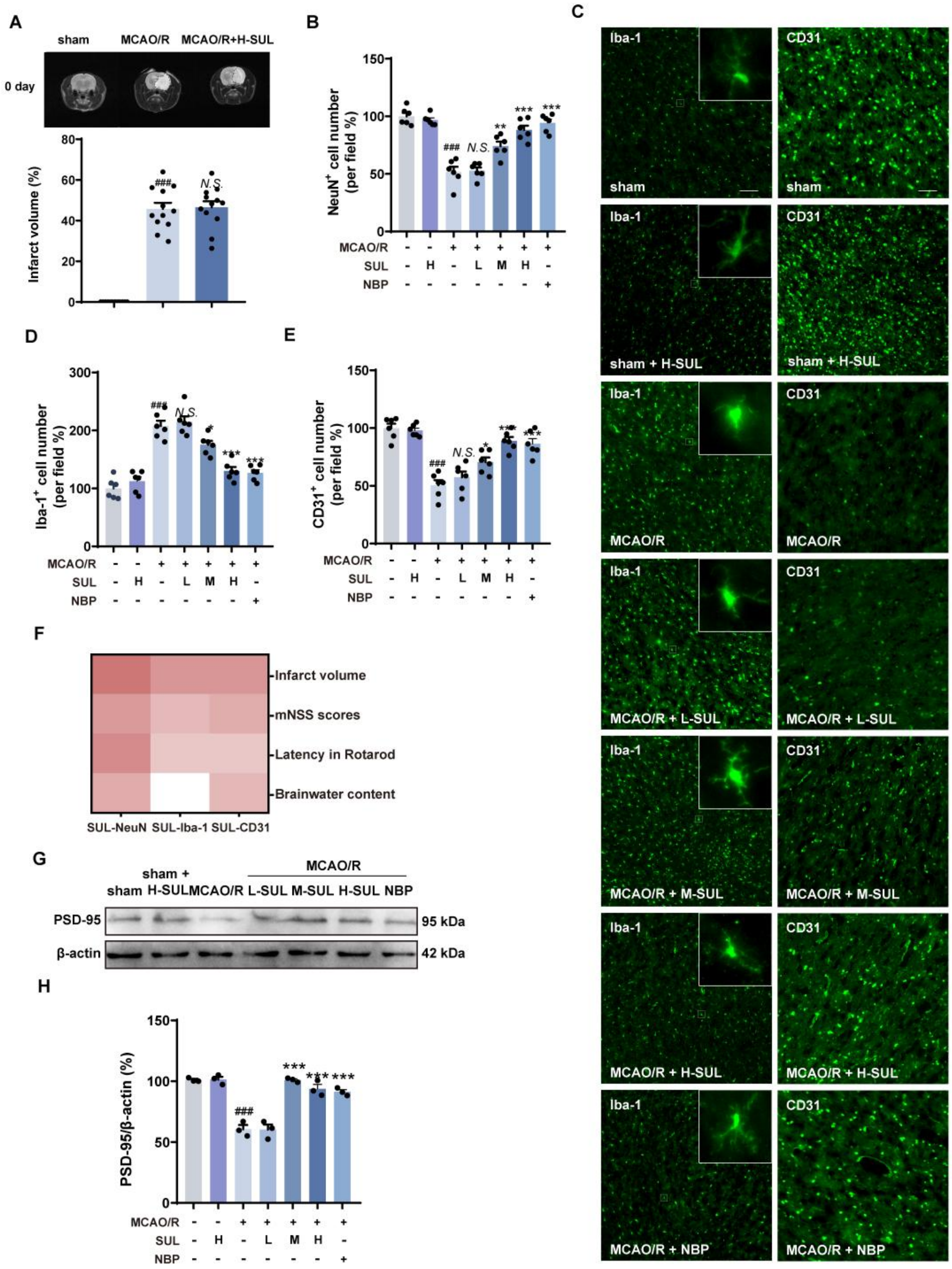

199

200

Supplementary Figure S5

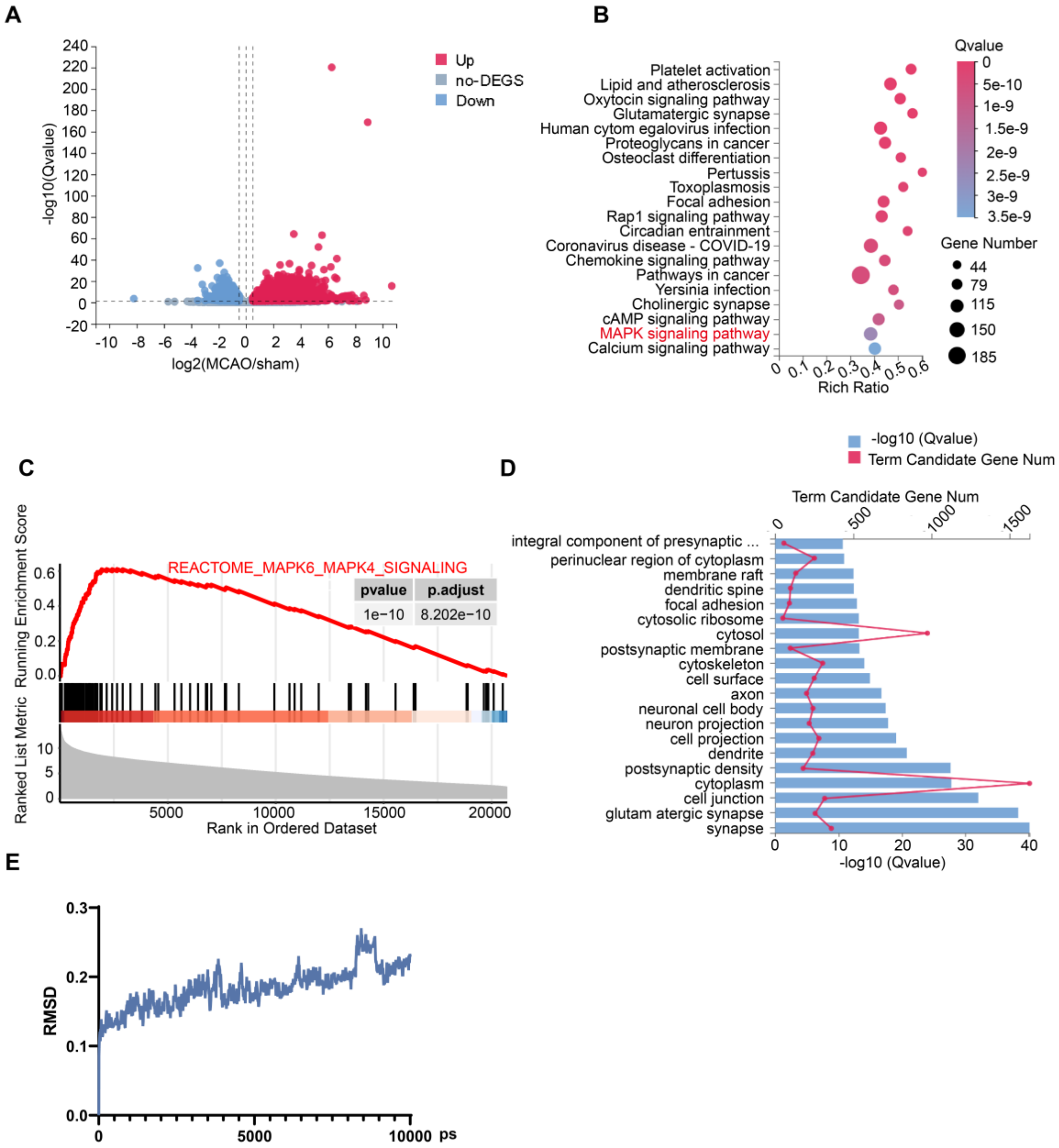

Supplementary Figure S6

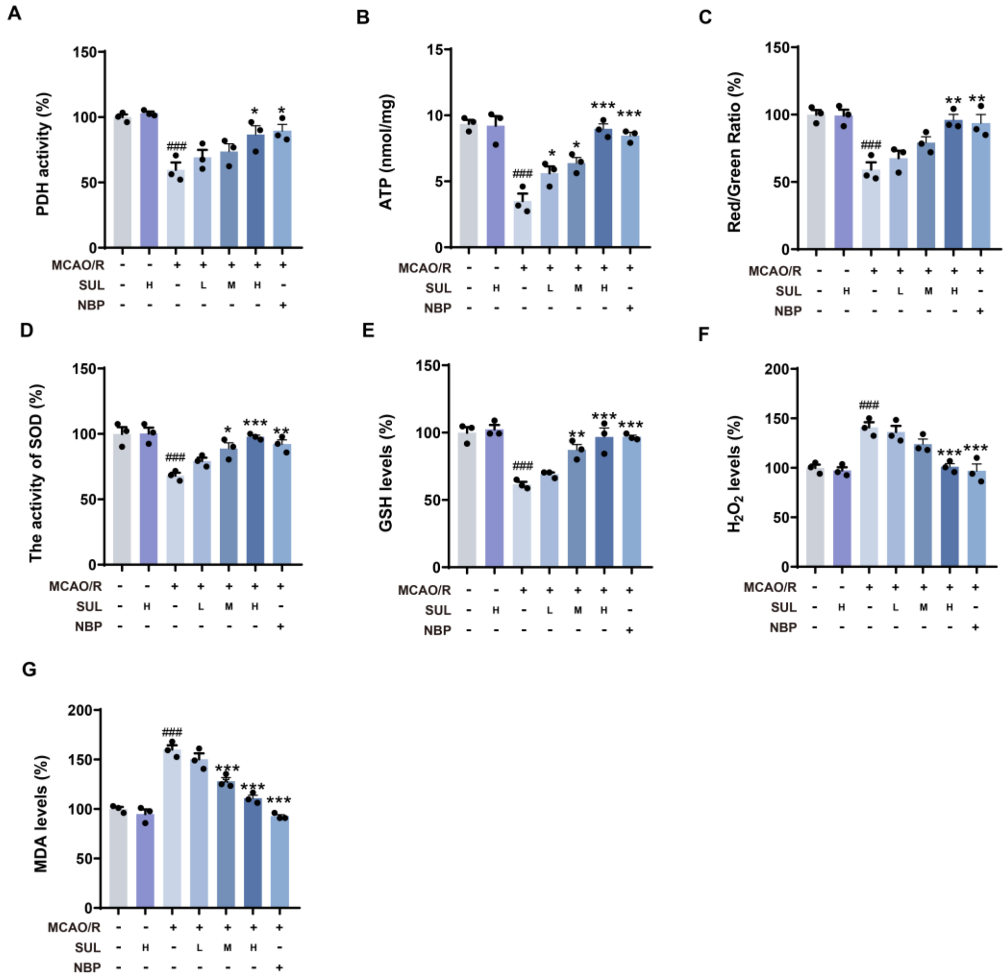

Supplementary Table S1. Abbreviation list

**Abbreviation**	**Full Name**
ANOVA	Analysis of Variance
Avalon	Avalon Fingerprint
ATP	Adenosine Triphosphate
Bax	Bcl-2-associated X protein
BCA	Bicinchoninic Acid Assay
Bcl-2	B-cell lymphoma 2
CCCP	Carbonyl Cyanide 3-Chlorophenylhydrazone
CETSA	Cellular thermal shift assay
CMap	Connectivity Map
DARTS	Drug Affinity Responsive Target Stability
DCFH-DA	2',7'-Dichlorodihydrofluorescein Diacetate
DEGs	Differentially Expressed Genes
DMSO	Dimethyl Sulfoxide
ECFP4	Extended Connectivity Fingerprints with radius = 2
ERK	Extracellular signal-regulated kinase
FCFP4	Functional-Class Fingerprints with radius = 2
I/R	Ischemia Reperfusion
GEO	Gene Expression Omnibus
GBDT	Gradient Boosting Decision Tree
GSH	Glutathione

KEGG	Kyoto Encyclopedia of Genes and Genomes
LIMMA	Linear Models for Microarray Data
MACCS	Molecular Access System Keys
MAPK	Mitogen-Activated Protein Kinase
MAE	Mean Absolute Error
MCAO/R	Middle Cerebral Artery Occlusion/Reperfusion
MDA	Malondialdehyde
MolWt	Molecular Weight
MPER	Mammalian Protein Extraction Reagent
MRE	Mean Relative Error
MSE	Mean Squared Error
mtDNA	Mitochondrial DNA
NBP	N-Butylphthalide
OGD/R	Oxygen-Glucose Deprivation/Reperfusion
p38	p38 mitogen-activated protein kinase
p-p38	Phosphorylated p38 mitogen-activated protein kinase
p-ERK	Phosphorylated Extracellular Signal-Regulated Kinase
PDK2	Pyruvate Dehydrogenase Kinase 2
PDH	Pyruvate Dehydrogenase
p-JNK	Phosphorylated c-Jun N-terminal Kinase
QED	Quantitative Estimate Of Drug-Likeness
Rdkit	A collection of cheminformatics and machine learning tools
RF	Random Forest

RIPA	Radio Immunoprecipitation Assay
RMSE	Root Mean Squared Error
ROS	Reactive Oxygen Species
RNA seq	RNA sequencing
SHAP	SHapley Additive exPlanations
SMILES	Simplified Molecular Input Line Entry System
siRNA	Small Interfering RNA
SOD	Superoxide Dismutase
SPR	Surface Plasmon Resonance
SVM	Support Vector Machine
TCA	Tricarboxylic Acid
TUNEL	Terminal Deoxynucleotidyl Transferase-mediated dUTP Nick-End Labeling
TPSA	Topological Polar Surface Area

Supplementary Table S2. The model performance comparison results for different molecular fingerprints based on GBDT

Model_metrics	Avalon	ECFP4	FCFP4	MACCS	RDKit	Chempy
Train_MRE	0.86%	0.17%	0.54%	1.08%	0.55%	0.40%
Train_MAE	0.05	0.01	0.03	0.06	0.03	0.02
Train_MSE	0.0046	0.0035	0.0062	0.0142	0.0077	0.0015
Train_RMSE	0.0676	0.0589	0.0785	0.1192	0.0879	0.0385
Train_R^2	0.9906	0.9929	0.9874	0.9709	0.9842	0.9970
Test_MRE	6.56%	5.50%	5.66%	5.86%	5.27%	4.88%
Test_MAE	0.34	0.30	0.29	0.31	0.27	0.26
Test_MSE	0.2022	0.1623	0.1520	0.2020	0.1160	0.1113
Test_RMSE	0.4497	0.4029	0.3899	0.4495	0.3406	0.3336
Test_R^2	0.7714	0.8165	0.8281	0.7716	0.8688	0.8742

Supplementary Table S3. The model performance comparison results for different molecular fingerprints based on SVM

Model_metrics	Avalon	ECFP4	FCFP4	MACCS	RDKit	Chempy
Train_MRE	2.16%	1.77%	1.89%	2.41%	1.97%	2.48%
Train_MAE	0.12	0.09	0.10	0.13	0.10	0.13
Train_MSE	0.0234	0.0131	0.0147	0.0286	0.0175	0.0355
Train_RMSE	0.1529	0.1145	0.1211	0.1693	0.1324	0.1885
Train_R^2	0.9521	0.9731	0.9700	0.9413	0.9641	0.9272
Test_MRE	7.61%	7.39%	5.99%	6.65%	5.93%	7.61%
Test_MAE	0.40	0.39	0.31	0.34	0.30	0.42
Test_MSE	0.2962	0.2449	0.1773	0.2299	0.1462	0.3654
Test_RMSE	0.5442	0.4949	0.4211	0.4795	0.3824	0.6045
Test_R^2	0.6652	0.7231	0.7995	0.7401	0.8347	0.5869

Supplementary Table S4. The model performance comparison results for different molecular fingerprints based on RF

Model_metrics	Avalon	ECFP4	FCFP4	MACCS	RDKit	Chempy
Train_MRE	2.53%	3.16%	3.07%	3.29%	3.06%	2.88%
Train_MAE	0.14	0.18	0.17	0.18	0.17	0.16
Train_MSE	0.0477	0.0630	0.0681	0.0635	0.0633	0.0621
Train_RMSE	0.2184	0.2510	0.2610	0.2521	0.2517	0.2492
Train_R2	0.9023	0.8710	0.8605	0.8699	0.8703	0.8729
Test_MRE	5.52%	6.04%	5.82%	6.24%	5.92%	5.59%
Test_MAE	0.29	0.33	0.32	0.34	0.32	0.30
Test_MSE	0.1371	0.1797	0.1829	0.2169	0.1790	0.1574
Test_RMSE	0.3703	0.4239	0.4276	0.4657	0.4231	0.3967
Test_R2	0.8450	0.7969	0.7933	0.7548	0.7976	0.8221

Supplementary Table S5. The hyper-parameter for three models optimized by TPE

Model_type	Main hyper-parameter	Space	Step	Distribution	Optimal value
RF	n_estimators	[10, 50]	5	Quniform	10
	max_depth	[5, 20]	1	Quniform	13
	max_features	[5, 30]	5	Quniform	25
SVM	kernel	[rbf, sigmoid, poly]	-	Categorical	rbf
	shrinking	[Ture, False]	-	Categorical	True
	C	[0.001, 1000]	-	Uniform	997.35
	gamma	[0.0001, 8] or 1/features	-	Uniform	1/features
GBDT	n_estimators	[50,100]	5	Quniform	75
	max_depth	[5, 20]	1	Quniform	7
	learning_rate	[0.05,0.15]		Uniform	0.11
	subsample	[0.7, 1.0]	0.1	Uniform	0.8

n_estimators is the number of decision trees, max_depth is the maximum depth of decision trees, max_features is the maximum number of features for constructing decision trees, Shrinking is whether to use a shrinking heuristic search method, C is

regularization term, kernel is Kernel function, Gamma is the bandwidth parameter, learning_rate is the learning step size, subsample is the proportion of subsample to avoid over-fitting under the condition of unbalanced samples.

Supplementary Table S6. Rat grouping and numbers

First Experiment

Detection Item	Sham	Sham+H-SUL	MCAO/R	MCAO/R +L-SUL	MCAO/R +M-SUL	MCAO/R +H-SUL	MCAO/R +NBP	Notes
Initial Number	12	12	12	12	12	12	12	All groups had consistent baselines
7-day Survival	12	12	8	8	9	10	10	Within 7 days after surgery, 9 rats died, and 6 rats were excluded due to behavioral scores not meeting the criteria
Brain Water/TTC/mNSS/Rotarod test	6	6	6	6	6	6	6	Randomly selected from surviving individuals (using a random number generator)
Immunofluorescence	6	6	2	2	3	4	4	

Second Experiment

Detection Item	Sham	Sham+H-SUL	MCAO/R	MCAO/R +L-SUL	MCAO/R +M-SUL	MCAO/R +H-SUL	MCAO/R +NBP	Notes
Initial Number	12	12	12	12	12	12	12	
7-day Survival	12	12	7	7	8	11	10	5 rats died, and 12 rats were excluded due to not meeting the criteria.
MRI Detection	12	–	7	–	–	11	–	"–" indicates no detection (as the efficacy of SUL was already verified in the first experiment)
Transcriptome	3	–	3	–	–	3	–	Three rats in the MCAO/R group were shared with WB and kits
WB/Kit	3	3	3	3	3	3	3	Randomly selected from surviving individuals
Immunofluorescence	–	–	4	4	3	2	2	Randomly selected from surviving individuals
Remaining	6	9	0	0	2	3	5	Used for preliminary experiments

Supplementary Table S7. Neurological function score

Trial projects	Scoring Criteria
Motor tests	
Raising rat by tai (0-3)	
Fore limb flexion	1
Hind limb flexion	1
Head moved >10° to vertical axis within 30 s	1
Placing rat on floor (0-3)	
Normal walking	0
Unable to go straight	1
Turning in a circle to the paralyzed side	2
Falls down to paretic side	3
Sensory tests (0-2)	
Orientation test (visual and tactile test, mouse hand moves slowly to edge of table, front paw rests very slowly on edge of table or does not bend)	1
Proprioception tests (depth perception, pressing the mouse to the edge of the table, stimulating the muscles of the extremities that have lost resistance)	1
Beam balance tests (0-6)	
Balance, postural stability	0
Take one side of the beam	1
A leg falls off the beam while holding it	2
Hold, drop or spin limbs off the beam (>60 s)	3
Attempts to maintain balance but eventually falls (>40 s)	4
Attempts to maintain balance but eventually falls (>20 s)	5

Full Fall: falls or rises from a beam without moving (<20 s)	6
Reflexes and irregular movements	
Pinna reflex (head shake when auditory meatus is touched)	1
Corneal reflex (head shake when cotton is lightly touched to the cornea)	1
Startle reflex (motor response to vocal stimuli formed by fast-	1
bouncing cardboard)	1
Seizures, myoclonus, myodystony	
Maximum points	18

Supplementary Table S8. Data statistical methods and confidence intervals

Figure	Statistical methods	Confidence intervals
2K	One-way ANOVA	F (20,42) = 51.970, p < 0.001
2M	One-way ANOVA	F (6,14) = 106.558, p < 0.001
3B	Survival analysis	Sham vs. MCAO/R: Chi-square = 10.93, df = 1, P = 0.0009<br>MCAO/R vs. MCAO/R+L-SUL: Chi-square = 4.82, df = 1, P = 0.9945<br>MCAO/R vs. MCAO/R+M-SUL: Chi-square = 0.41, df = 1, P = 0.523<br>MCAO/R vs. MCAO/R+H-SUL: Chi-square = 3.90, df = 1, P = 0.0484<br>MCAO/R vs. MCAO/R+NBP: Chi-square = 2.51, df = 1, P = 0.1131
3C	ANOVA with repeated measurements	Bas: F (6,35) = N.A.<br>D0: F (6,35) = 121.711, p < 0.001<br>D1: F (6,35) = 62.125, p < 0.001<br>D3: F (6,35) = 40.298, p < 0.001<br>D7: F (6,35) = 26.790, p < 0.001

3D	ANOVA with repeated measurements	Bas: F (6,35) = 1.552, p = 0.191.<br>D0: F (6,35) = 106.763, p < 0.001<br>D1: F (6,35) = 76.354, p < 0.001<br>D3: F (6,35) = 20.258, p < 0.001<br>D7: F (6,35) = 19.407, p < 0.001
3F	ANOVA with repeated measurements	D1: F (2,25) = 151.407, p < 0.001<br>D3: F (2,25) = 51.009, p < 0.001<br>D7: F (2,25) = 19.869, p < 0.001
3H	One-way ANOVA	F (6,35) = 78.224, p < 0.001
3I	One-way ANOVA	F (6,35) = 10.425, p < 0.001
3M	One-way ANOVA	F (6,14) = 18.691, p < 0.001
3N	One-way ANOVA	F (6,14) = 45.335, p < 0.001
3O	One-way ANOVA	F (6,14) = 134.613, p < 0.001
4F	One-way ANOVA	F (6,14) = 15.564, p < 0.001
4G	One-way ANOVA	F (6,14) = 1145.413, p < 0.001
4H	One-way ANOVA	F (6,14) = 820.467, p < 0.001
4J	One-way ANOVA	F (6,14) = 225.116, p < 0.001
4K	One-way ANOVA	F (6,14) = 158.419, p < 0.001
4L	One-way ANOVA	F (6,14) = 400.728, p < 0.001
4M	One-way ANOVA	F (8,18) = 15.604, p < 0.001
5F	One-way ANOVA	F (5,12) = 46.639, p < 0.001
5G	One-way ANOVA	F (5,12) = 126.937, p < 0.001
5H	One-way ANOVA	F (5,12) = 286.896, p < 0.001

6B	One-way ANOVA	$F(5,12) = 122.790$, $p < 0.001$
6C	One-way ANOVA	$F(6,14) = 51.029$, $p < 0.001$
6D	One-way ANOVA	$F(5,12) = 9.318$, $p < 0.001$
6E	One-way ANOVA	$F(6,14) = 15.757$, $p < 0.001$
6F	One-way ANOVA	$F(5,12) = 47.794$, $p < 0.001$
6G	One-way ANOVA	$F(5,12) = 14.190$, $p < 0.001$
6I	One-way ANOVA	$F(6,14) = 192.804$, $p < 0.001$
6L	One-way ANOVA	$F(2,6) = 6.637$, $p < 0.001$
6M	One-way ANOVA	$F(2,6) = 217.417$, $p < 0.001$
7C	One-way ANOVA	$F(6,14) = 755.798$, $p < 0.001$
7D	One-way ANOVA	$F(6,14) = 241.201$, $p < 0.001$
7E	One-way ANOVA	$F(2,6) = 142.903$, $p < 0.001$
7F	One-way ANOVA	$F(5,12) = 131.052$, $p < 0.001$
7G	One-way ANOVA	$F(6,14) = 57.875$, $p < 0.001$
7H	One-way ANOVA	$F(6,14) = 48.417$, $p < 0.001$
7I	One-way ANOVA	$F(6,14) = 352.043$, $p < 0.001$
7J	One-way ANOVA	$F(6,14) = 265.530$, $p < 0.001$
7K	One-way ANOVA	$F(5,12) = 73.048$, $p < 0.001$
7L	One-way ANOVA	$F(5,12) = 118.694$, $p < 0.001$
7M	One-way ANOVA	$F(5,12) = 122.827$, $p < 0.001$
7N	One-way ANOVA	$F(5,12) = 110.074$, $p < 0.001$
8B	One-way ANOVA	$F(5,12) = 56.837$, $p < 0.001$
8C	One-way ANOVA	$F(5,12) = 57.881$, $p < 0.001$
8E	One-way ANOVA	$F(5,12) = 30.903$, $p < 0.001$
8F	One-way ANOVA	$F(5,12) = 59.534$, $p < 0.001$

8G	One-way ANOVA	F (5,12) = 46.936, p < 0.001
8I	One-way ANOVA	F (5,12) = 531.884, p < 0.001
8J	One-way ANOVA	F (5,12) = 28.410, p < 0.001
8K	One-way ANOVA	F (5,12) = 756.532, p < 0.001
S3A	One-way ANOVA	F (5,12) = 34.016, p < 0.001
S3B	One-way ANOVA	F (5,12) = 20.316, p < 0.001
S3C	One-way ANOVA	F (5,12) = 36.844, p < 0.001
S3D	One-way ANOVA	F (5,12) = 48.625, p < 0.001
S3E	One-way ANOVA	F (5,12) = 28.285, p < 0.001
S3F	One-way ANOVA	F (5,12) = 26.480, p < 0.001
S3G	One-way ANOVA	F (5,12) = 75.184, p < 0.001
S3H	One-way ANOVA	F (5,12) = 13.943, p < 0.001
S3I	One-way ANOVA	F (5,12) = 94.334, p < 0.001
S3J	One-way ANOVA	F (5,12) = 150.640, p < 0.001
S3K	One-way ANOVA	F (5,12) = 32.764, p < 0.001
S3L	One-way ANOVA	F (5,12) = 20.138, p < 0.001
S3M	One-way ANOVA	F (5,12) = 21.467, p < 0.001
S3N	One-way ANOVA	F (5,12) = 30.330, p < 0.001
S3O	One-way ANOVA	F (5,12) = 64.561, p < 0.001
S3P	One-way ANOVA	F (5,12) = 69.987, p < 0.001
S3Q	One-way ANOVA	F (5,12) = 54.049, p < 0.001
S3R	One-way ANOVA	F (5,12) = 23.480, p < 0.001
S3S	One-way ANOVA	F (5,12) = 41.405, p < 0.001
S4A	One-way ANOVA	sham *vs.* MCAO/R p < 0.001
		sham *vs.* H-SUL p < 0.001

		H-SUL *vs.* MCAO/R p = 0.969
S4B	One-way ANOVA	F (6,35) = 37.049, p < 0.001
S4D	One-way ANOVA	F (6,35) = 37.542, p < 0.001
S4E	One-way ANOVA	F (6,35) = 24.391, p < 0.001
S4H	One-way ANOVA	F (6,35) = 46.044, p < 0.001
S6A	One-way ANOVA	F (6,14) = 10.496, p < 0.001
S6B	One-way ANOVA	F (6,14) = 22.059, p < 0.001
S6C	One-way ANOVA	F (6,14) = 11.227, p < 0.001
S6D	One-way ANOVA	F (6,14) = 11.172, p < 0.001
S6E	One-way ANOVA	F (6,14) = 19.482, p < 0.001
S6F	One-way ANOVA	F (6,14) = 15.255, p < 0.001
S6G	One-way ANOVA	F (6,14) = 48.155, p<0.001

Bas: baseline, D0: 0 days after MCAO/R surgery, D1: 1 days after MCAO/R surgery, D3: 3 days after MCAO/R surgery, D7: 7 days after MCAO/R surgery, S3A: Supplementary figure 3A.
